# APAs Constraints to Voluntary Movements: The Case for Limb Movements Coupling

**DOI:** 10.3389/fnhum.2017.00152

**Published:** 2017-03-31

**Authors:** Fausto G. Baldissera, Luigi Tesio

**Affiliations:** ^1^RetiredMilan, Italy; ^2^Department of Biomedical Sciences for Health, Università degli Studi di MilanoMilan, Italy; ^3^Department of Neuro-Rehabilitation Sciences, Istituto Auxologico Italiano-IRCCSMilan, Italy

**Keywords:** APAs, limb movements coupling, in phase and antiphase coupling, direction principle, APAs destabilizing effects on coupling, coupled movements synchronization, motor learning/training

## Abstract

When rhythmically moving two limbs in either the same or in opposite directions, one coupling mode meets constraints that are absent in the other mode. Isodirectional (ISO) flexion-extensions of the ipsilateral hand and foot can be easily performed with either the hand prone or supine. Instead, antidirectional (ANTI) movements require attentive effort and irresistibly tend to reverse into ISO when frequency increases. Experimental evidence indicates that the direction dependent easy-difficult dichotomy is caused by interference of the anticipatory postural commands associated to movements of one limb with voluntary commands to the other limb. Excitability of the resting wrist muscles is subliminally modulated at the period of ipsilateral foot oscillations, being phase-opposite in the antagonists and distributed so as to facilitate ISO and obstacle ANTI coupling of the hand (either prone or supine) with the foot. Modulation is driven by cortical signals dispatched to the forearm simultaneously with the voluntary commands moving the foot. If right foot oscillations are performed when standing on the left foot with the right hand touching a fixed support, the subliminal excitability modulation is replaced by overt contractions of forearm muscles conforming the APAs features. This suggests that during hand-foot ANTI coupling the voluntary commands to forearm muscles are contrasted by APAs commands of opposite sign linked to foot oscillations. Correlation between the easy-difficult dichotomy and the APAs distribution is also found in coupled adduction-abduction of the arms or hands in the transverse plane and in coupled flexion-extension of the arms in the parasagittal plane. In all these movements, APAs commands linked to the movement of each limb reach the motor pathways to the contralateral muscles homologous to the prime movers and can interfere during coupling with their voluntary activation. APAs are also generated in postural muscles of trunk and lower limbs and size-increase when the movement frequency is incremented. The related increase in postural effort apparently contributes in destabilizing the difficult coupling mode. Motor learning may rely upon more effective APAs. APAs and focal contraction are entangled within the same voluntary action. Yet, neural diseases may selectively impair APAs, which represent a potential target for rehabilitation.

## Introduction

This review is concerned with a so far neglected aspect of the interaction between Anticipatory Postural Adjustments, APAs, and voluntary movements. Beside the APAs main function of assisting the execution of voluntary movements of any particular body segment, under defined circumstances APAs may result in the impairment of body movements. In particular, this occurs during coupled movements of the limbs and is especially apparent in rhythmic cyclic movements.

In many gestures of everyday life, e.g., handiworks, sport activities or music playing, the voluntary movements of different limb segments may be coupled into a variety of combinations. It is common experience that when oscillating a given couple of limbs in either the same or in opposite directions (in external coordinates), movements are easily performed in one coupling mode whereas coupling in the other mode is hampered. The evidence summarized here strongly suggests that the constraints selectively hindering one specific coupling mode, either the iso- or the antidirectional one, are generated by the APAs associated with the same voluntary limb movements.

The review is structured as follows. Sections *The “Direction Principle” in Coupling Flexion-Extension of the Ipsilateral Hand and Foot. Easy vs. Difficult Coupling Modes* and *Role of Kinesthetic Afferents from the Moving Segments in Controlling the Hand-Foot Synchronism during Coupled Movements: An Interlimb or Intralimb Feedback?* will describe the features of the hand-foot coupled movements and the mechanisms providing the control of movements synchronization. In turn, Sections *Neural Mechanisms Underlying the “Direction Principle” in Hand-Foot Coupling* and *Role of APAs in Differentiating ISO vs. ANTI Coupling Modes in Other Types of Limb Movements* will discuss experimental evidence of the selective APAs effects on coupling coordination in the above as well as in other couples of limbs. Section *Postural Constraints from Neuroscience to Sports and Rehabilitation Medicine* will discuss some potential implications of this knowledge in motor learning, force training, and rehabilitation.

### Direction-dependent differential coordination of coupled movements of the limbs

The early observations on limb movements coupling [Müller, 1840; Meige, 1901; Noica (Bucarest), 1912] report that, when performing mirror movements like drawing circles in the air with the two hands in the parasagittal plane, it is quite easy to rotate the hands in the same direction, whereas circling the hands in opposite directions is difficult or even impossible.

For several decades this phenomenon did not attract further interest. On occasion of a seminar held by the first author over 30 years ago, Pietro Civaschi, a physiatrist at the Neurological Institute Carlo Besta in Milan, asked for some explanation about the striking preference of normal individuals to move two ipsilateral body segments together (for instance, the ipsilateral hand and foot in the parasagittal plane) in the same rather than in opposite directions. Having no explanation at that moment, the answer was to analyze the matter together, in the hope of gaining a deeper insight.

This review will summarize the results of the experimental series stemmed from that proposal. The successive steps of this research entered different fields of motor neurosciences and were reported in an irregular series of separate papers. Because of the time dispersion of the reports and the variety of methodological and conceptual approaches utilized, it seemed convenient to facilitate the interested reader by tracing in one review the logic route connecting the successive experiments, interpretations and hypotheses.

**Figure 1:**
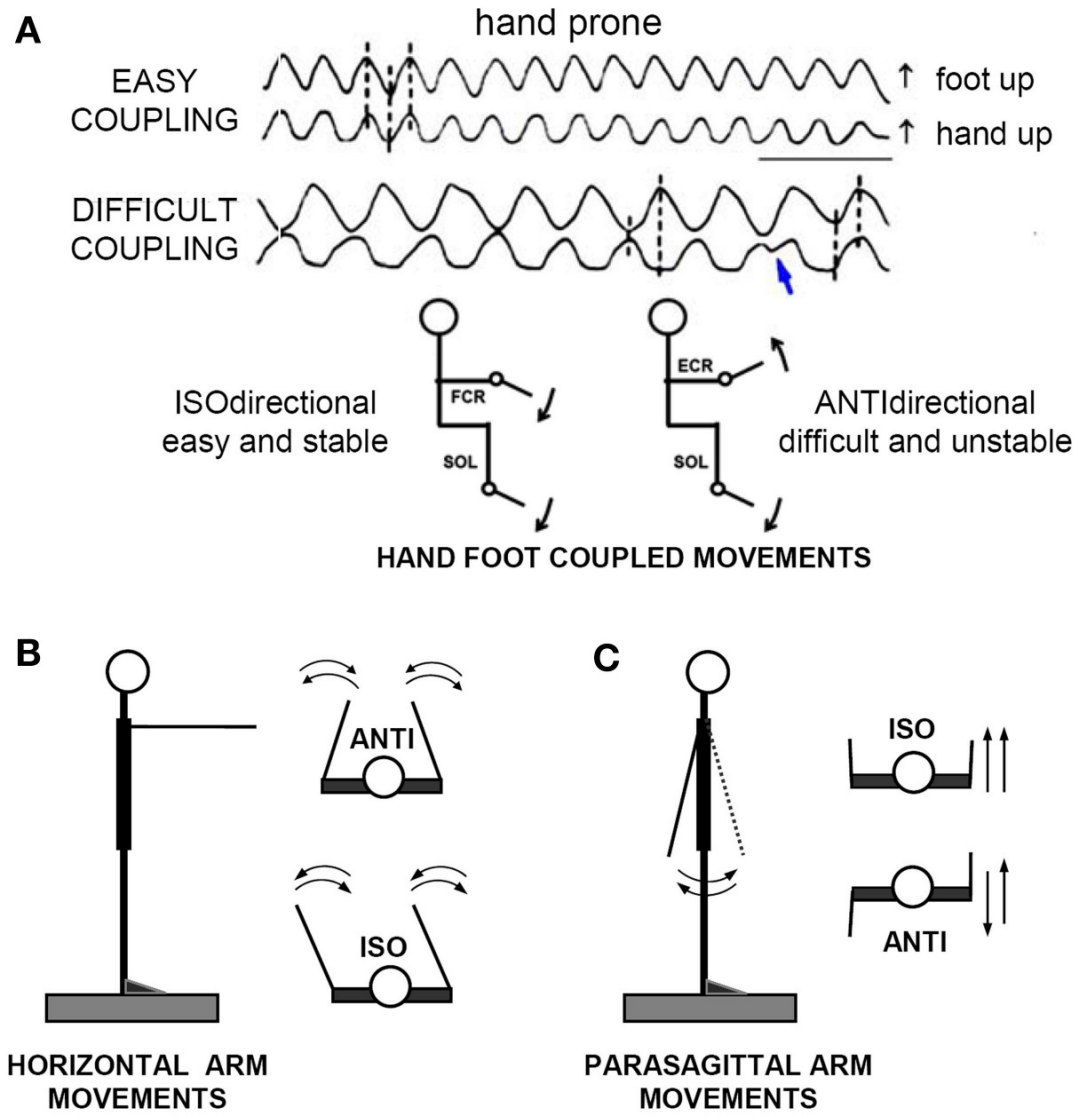
**The “direction principle” in hand-foot coupling. (A)** Subjects seated on an armchair, with the forearms supported in horizontal position, either prone, or supine. The right hand and foot were fixed to basculating supports, free to cover full-range flexion-extensions of the wrist and ankle. Despite their lower frequency, the ANTIdirectional (difficult) oscillations spontaneously reverse (blue arrow) to the easier ISOdirectional coupling. Time calibration, 1 s. **(B,C)** Schematic illustration of ISO and ANTI coupling of the arm cyclic movements in the horizontal and parasagittal plane, respectively. Reproduced from Baldissera et al. (1982) © Elsevier 1982, with permission of Elsevier.

Three different combinations of coupled limb movements were studied (Figure 1): (1) coupled flexion-extension of ipsilateral hand and foot in the parasagittal plane; (2) coupled adduction-abduction movements of the arms in the horizontal (transverse) plane and (3) coupled flexion-extension movements of the arms in the parasagittal plane.

The preferred coupling mode is isodirectional in movement types 1 and 3, and antidirectional in type 2. Substantial evidence was found that in all three movement types the direction-dependent dichotomy between preferred and non-preferred modes correlates with the distribution of the Anticipatory Postural Adjustments associated with the same movements.

For methodological, technical, and statistical details the original papers should be consulted.

## The “direction principle” in coupling flexion-extension of the ipsilateral hand and foot. easy vs. difficult coupling modes

When asked to perform simultaneous rhythmic flexions-extension movements of the extremities in the parasagittal plane, in the way they feel the most easy and spontaneous, all subjects normally choose to keep the forearm prone and to rotate the extremities in the same angular direction^1^ (isodirectional coupling, ISO), by synchronizing hand extension with foot dorsal flexion and hand flexion with foot plantar flexion (Figure 1A, uppermost traces).

If asked to increase the oscillations rate, all subjects are able to maintain the ISO coupling for more than 1 min even at the maximal possible rate (3–4 Hz). When requested to reverse the phase between the limbs (antidirectional coupling, ANTI, lower traces), the subjects generally stop briefly, restore the movements with some initial hesitation and/or errors, and are afterwards compelled to pay continuous attention to maintain the phase opposition. Any attention release, as well as any attempt to increase the movement frequency, eventually leads to a breaking point, beyond which the association reverses to the isodirectional pattern (blue arrow). Transitions is abrupt or preceded by a progressive shift of the interlimb phase difference, and occurs after a time that progressively shortens as the movement frequency increases: continuing for more than 10 s is impossible beyond a critical frequency that ranges between 1.2 and 2.5 Hz in different individuals.

Finally, when invited to repeat the task with the hand supine, all subjects confirm their preference for ISO coupling, now associating hand flexion to foot dorsal flexion and hand extension to foot plantar flexion. The determinant factor of coupling preference seems therefore to be the movements reciprocal direction (*direction principle*, Baldissera et al., 1982) and not a stable pattern of parallel innervation of specific muscles in the forearm and the leg.

### Coupling coordination: accuracy and stability of the interlimb relative phase

In the following years studies on limb coupled movements increased in number and the “direction principle” was found to hold in many other types of coupled movements of ipsilateral limbs (cfr Baldissera et al., 1982, 1991, 1994, 2000; Baldissera and Cavallari, 2001; Kelso and Jeka, 1992; Carson et al., 1995; Jeka and Kelso, 1995; Swinnen et al., 1995; Serrien and Swinnen, 1998), bilateral limbs (cfr. Kelso, 1984; Carson, 1993; Swinnen et al., 1995; Serrien and Swinnen, 1998; Swinnen, 2002) and also different segments within one limb (for instance flexion-extension of the wrist with flexion-extension of the elbow, as earlier recognized by Kots et al., 1971). As a general rule, one given coupling mode, in some occasions ISO and in others ANTI, is easily performed, subjectively preferred and better co-ordinated; whereas the opposite mode is difficult to perform (thus, non-preferred) and less coordinated. *Easy* and *preferred*, as well as *difficult* and *non-preferred* will be used hereafter as synonyms.

Kelso's theoretical elaboration (Haken et al., 1985, see Presentation 1 in the Supplementary Material) assimilates the coupled oscillations of two limbs to those of a system of non-linear oscillators moving in one of two stable states (in phase or in phase opposition) with different levels of stability. This model stresses the centrality of the *interlimb relative phase*, ΔΦ, as being the collective variable that describes the qualitative changes in pattern coordination. In real movements the ΔΦ variability, i.e., the coupling instability, is expressed by its standard deviation, SDΔΦ. The latter is positively related to the oscillation frequency and it is higher in the difficult (non-preferred) compared to the easy (preferred) coupling mode. Based on this observation, when the movement frequency, and thus SDΔΦ, reaches to a critical level, the model shifts by 180° from the unstable to the stable state. Accordingly, the values of SDΔΦ indicate for each value of the movement frequency the degree of coupling instability, and thus the difficulty in contrasting the phase reversal.

### Mechanical differences between the limbs and neural compensation for their desynchronizing effect on coupled movements

In order to understand the mechanistic origin of the differences in coordination stability between the two coupling modes, two interlaced aspects needed to be analyzed. First, how synchronization between the limbs is achieved and how this is influenced by the limb mechanical properties; second, why the interlimb coordination varies when the coupling mode is reversed. A response to the first question is proposed in Section *Role of Kinesthetic Afferents from the Moving Segments in Controlling the Hand-Foot Synchronism during Coupled Movements: An Interlimb or Intralimb Feedback?*. In Sections *Neural Mechanisms Underlying the “Direction Principle” in Hand-Foot Coupling and Role of APAs in Differentiating ISO vs. ANTI Coupling Modes in Other Types of Limb Movements* evidence will be presented suggesting that the constraints to the “difficult” mode depend on the directional distribution of the anticipatory postural adjustments associated with the primary movements.

#### Mechanical characteristics of the hand and foot oscillations

The oscillations of the hand (Stark, 1968; Stiles et al., 1983; Lakie et al., 1984; Lehman and Calhoun, 1990) and foot (Hunter and Kearney, 1982) can be satisfactorily modeled by a 2nd-order system (a pendulum) with lumped parameters (mass, stiffness, and viscosity). During passive oscillations, the unloaded hand behaves like a simple mass (negligible elastic and viscous momenta) but when the wrist is pre-loaded it behaves like a mass-spring system (Lehman and Calhoun, 1990). Also during voluntary cyclic flexion-extension of the wrist (Bobet and Norman, 1990) and elbow (Viviani et al., 1976), the phase relationships between the movers EMGs and the joint position approximate those of an ideal pendulum.

Accordingly, should two limbs share identical mechanical properties and be moved by homologous muscles (e.g., flexion-extension of the two hands), a common rhythm generator sending one and the same motor command in parallel to both segments (Schmidt et al., 1979; Turvey et al., 1989) would obtain a correct coupling at all frequencies. If instead the two limbs have different mechanical features, as it is the case for the hand and foot, the oscillations of the two limbs induced by a parallel synchronous command will have different phase delays with respect to the command itself. Moreover, the interlimb phase difference would increase as the movement frequency is raised.

**Figure 2:**
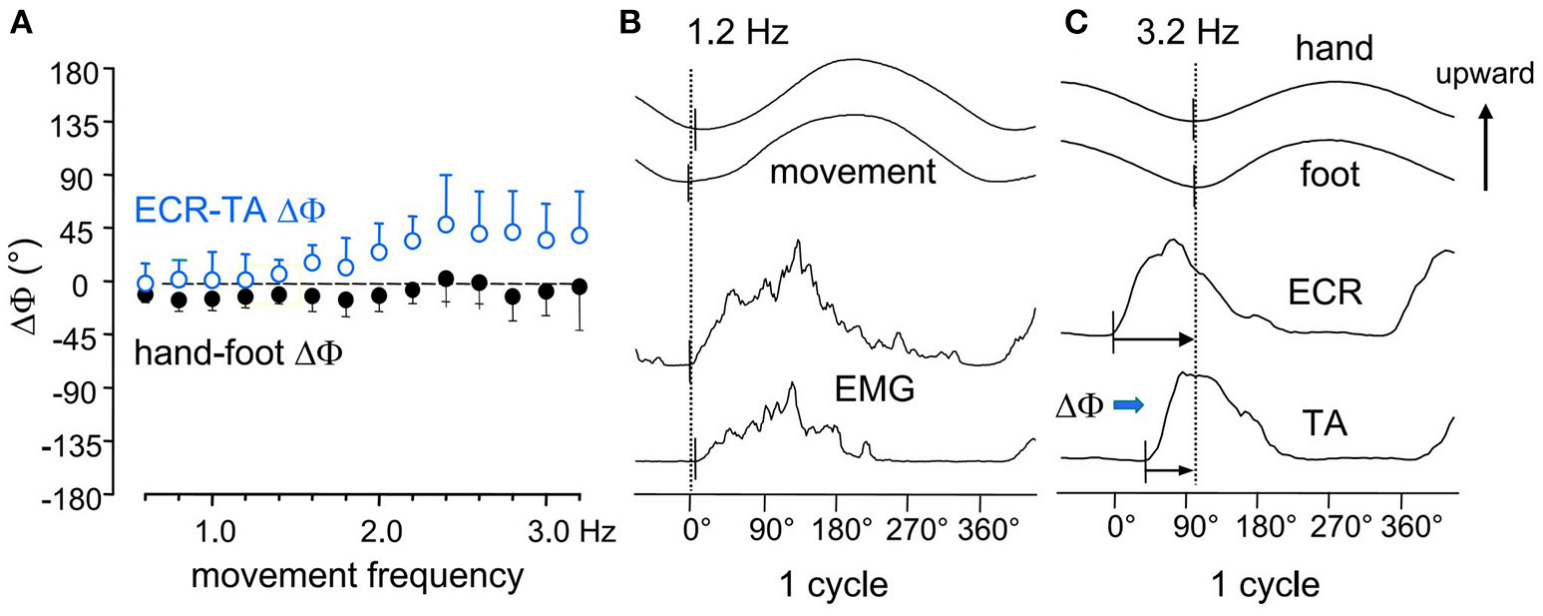
**(A)** the phase difference between the two movements (*hand-foot* ΔΦ, filled black circles) remains mostly unmodified over the entire frequency range. **(B)** at 1.2 Hz the upward rotation of the two limbs and the onset of the integrated EMGs in the respective movers *ECR, Extensor Carpi Radialis*; *TA, Tibialis Anterior*, all start synchronously. **(C)** at 3.2 Hz, both movements lag the EMG burst onsets, the hand to a larger extent than the foot (black arrows), revealing that the two limbs have different mechanical features. This difference, however, is compensated for by a calibrated advance of the ECR on the TA burst *(ECR-TA* ΔΦ, blue arrow) which increases with frequency (open circles in A) and maintains the movements synchronism. Reproduced from Baldissera et al. (2000), © Springer-Verlag Berlin Heidelberg 2000, with permission of Springer.

Based on the greater mass of the foot compared to the hand, one would predict that, when driven by a common command, the foot oscillations are phase-delayed with respect to the hand by an extent proportionate to the movement frequency. In fact (Baldissera et al., 2000), the hand movements cycle slightly lags the required synchronism with the foot cycle (namely 0° in ISO and 180° in ANTI) by an almost constant value over the entire frequency range (Figure 2A). Correspondingly (compare Figure 2B and Figure 2C), as the frequency is raised the onset of the EMG activity in forearm muscles (*Extensor Carpi Radialis*, ECR) progressively phase-advances the EMG onset in leg muscles (*Tibialis Anterior*, TA). This occurs in both coupling modes.

These findings would indicate that: (1) the mechanical impedance is greater for the hand than for the foot, despite the smaller mass of the hand; and (2) when the movement frequency is raised, the increase of the hand-foot delay is avoided by a progressive phase advance of the EMG burst in ECR with respect to TA, demonstrating that a neural mechanisms has intervened to counteract the desynchronizing effects of the mechanical disparities.

In conformity with the afore mentioned pendulum model (see features and details in Presentation 2 in Supplementary Material), the input-output phase relations of each limb were derived (Baldissera et al., 2000, 2004; Esposti et al., 2005) from the frequency-dependent changes of the phase delay between the onset of the EMG activity in the movement prime movers (input) and the homologous instant of the related movements (output).

The frequency responses of both the hand and the foot fit the pendulum equation. The foot response, however, shows a slower decay and a higher value of the ratio √*K*/*I* (the resonant frequency) compared to the corresponding response of the hand, indicating that the larger mass of the foot is naturally associated with an even larger active stiffness. This difference will justify the smaller EMG-movement lag in the foot than the in hand illustrated in Figure 2C.

## Role of kinesthetic afferents from the moving segments in controlling the hand-foot synchronism during coupled movements: an interlimb or intralimb feedback?

The neural control mechanisms that compensates for the desynchronizing effects of the mechanical disparities between the limbs might theoretically monitor the deviations of the instantaneous hand-foot relative phase, ΔΦ, from the programmed value (0° or 180°) and use these error signal for recovering the intended synchronism. Alternatively, each oscillating limb might be provided with an independent neural control which monitors the position of that limb and reacts so as to eliminate any phase mismatch between the rhythmic central command and the actual movement. The functional difference between these two mechanisms lies in the fact that the first one would tend to keep the interlimb relative phase constant whilst being unable to control the synchronization of the limbs oscillations with the central rhythm generator; while the second mechanism would simultaneously achieve both results. Distinguishing between these two mechanisms was attempted by two complementary approaches.

First, the existence of a crossed kinesthetic feedback between the limbs was explored by testing whether the afferent signals generated during voluntary oscillations of one limb may induce excitability changes in the motor structures innervating the second, resting limb. Second, the phase synchronization of the hand and foot oscillations with an external clock signal was measured both when the two segments were moved in isolation and when they were coupled, either iso- or antidirectionally. This allowed to test whether the clock-movement phase-delay, measured when one limb is moved alone, changed as the two limbs were coupled, thus proving that one limb can influence the other in controlling the interlimb synchronization.

### Modulation of motoneuronal excitability in the resting forearm muscles during voluntary oscillations of the ipsilateral foot

The excitability of wrist flexor and extensor muscles in the resting forearm was tested during voluntary oscillations of the ipsilateral foot, so as to ascertain whether it was modified by the foot movements (Baldissera et al., 2002).

**Figure 3:**
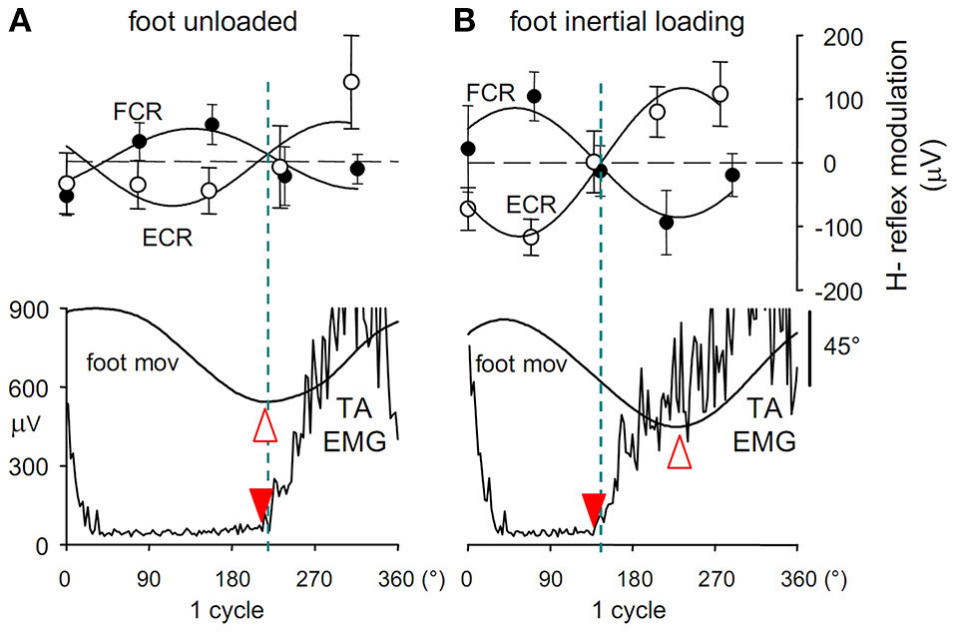
**(A)** Cyclic modulation the H-reflex size, phase-opposite in the two forearm antagonists *ECR* (open circles) and *FCR* (filled circles), at 5 delays during ipsilateral foot oscillations.Modulation is expressed as the absolute deviation of the reflex size from the cycle mean value and is plotted together with the angular position of the ipsilateral foot (*foot mov*, dorsal flexion up) and the EMG activity in TA muscle (*TA EMG*). Best-fit sinusoidal functions (solid lines) with the same period as movement are superimposed to the H-reflex plots. Crossing between the two H modulation sine waves (dashed blue line) occurs almost synchronously with both the foot flexion peak (open arrowhead) and the onset of TA activation (filled arrowhead). After foot inertial loading **(B)** the foot flexion peak lags the onset of the TA burst by about 90°, while the H-reflex modulation remains phase-linked to the TA activation, showing to be insensitive to the afferent signals monitoring the phase shift of the foot position. Figure assembled with data from Cerri et al. (2003) and Borroni et al. (2004).

Indeed, during foot oscillations the size of the H-reflex evoked in the resting *Flexor Carpi Radialis* (FCR) undergoes a sine-wave modulation with the same period as the foot movements (Figure 3A). With the forearm in prone position, the FCR H-reflex (filled circles) is facilitated during foot plantar flexion and dis-facilitated during dorsiflexion, while a phase-opposite H-modulation is seen in the antagonist *Extensor Carpi Radialis*, ECR (open circles). Moreover, when the forearm position is changed from prone to supine, the modulation in both ECR and FCR shifts by 180° with respect to the foot movement, thus maintaining the phase opposition in the two antagonists (Borroni et al., 2004; Byblow et al., 2007).

Should these subliminal excitability changes in forearm flexor and extensor motoneurons occur when the hand is moved together with the foot, they would have the same sign (excitatory or inhibitory) as the simultaneous voluntary commands to forearm muscles during ISO coupling; and an opposite sign in ANTI coupling, thus favoring the former and contrasting the latter coupling mode. Moreover, this pattern would hold both when the hand is prone and when it is supine, in agreement with the “direction principle” of hand-foot coupling.

The compound muscle action potentials (CMAPs) evoked in FCR and ECR muscles by transcranial magnetic stimulation (TMS) of the primary motor cortex are also modulated in size during foot oscillations, with the same time course of, but more strongly than, the H-reflex. Finally, interactions between the corticospinal and the Ia afferent effects (Baldissera et al., 2002) convincingly prove that the changes in motoneurons excitability associated with foot oscillations have a cortical origin.

Similar results were also obtained by other investigators in the context of bimanual coupling. In sitting subjects with the hands in semiprone position, Carson et al. (2004) found that the amplitude of the H-reflex and CMAPs in the resting FCR muscle is cyclically modulated during rhythmic oscillations of the contralateral wrist in the horizontal plane. Further, by analysing the EMG spectrogram, Ridderikhoff et al. (2005) found evidence for bursting activity in the resting ECR that occurs in phase with the extension of the contralateral wrist. Also in these cases the excitability modulation would favor the preferred coupling mode, which is mirror symmetrical (ANTI in external coordinates) in these hand movements.

Altogether, these results seemingly supported the working hypothesis that the kinesthetic signals for position (and/or velocity) from one limb cyclically activate the corticospinal projections to the other limb, thereby modulating the motoneuron excitability. Through this mechanism, the afferent signals from the foot might in fact interfere—either positively or negatively according to the coupling mode—with the generation of the voluntary cortical commands to the hand.

This conclusion was soon falsified, however. Since application of an inertial load to the foot, as well as raising the frequency of foot movements, both increase the phase-delay of the foot oscillations with respect to the motor commands (Section *The “Direction Principle” in Coupling Flexion-Extension of the Ipsilateral Hand and Foot. Easy vs. Difficult Coupling Modes* and Presentation 2 in Supplementary Material), it was argued that if the H-reflex modulation at the forearm was driven by the kinesthetic information from the moving foot, if would remain phase linked to the foot rotation. Instead (Cerri et al., 2003), after both loading (Figure 3B) and/or increasing the oscillation frequency, the H-modulation in the forearm muscles remains tightly linked to the voluntary motor command, thus anticipating the foot movement. This finding disproves that the cyclic excitability modulation in forearm motoneurons is elicited by the afferent signals that transduce the foot rotation and suggests that it is instead generated in the forearm area of the primary motor cortex simultaneously with the voluntary commands dispatched to the foot. The view that the interlimb relative phase is controlled through an interlimb afferent feedback is then seriously challenged. The possibility remains, however, that the hypothesized crossed feedback was hidden in this experiment because it is only activated when both limbs are oscillated together.

If this were to be the case, however, the different mechanical properties of the two segments would imply that, when each segment is moved alone, the movement phase delay with respect to an external clock (*clock-mov delay)* is different in each limb, and that such difference increases as the oscillation frequency is raised. Consequently, achieving the movements synchronization during coupling through a crossed kinesthetic feedback would necessarily modify the “intrinsic” *clock mov-delay* of either one of the two limbs or both.

### Independent position control of each limb by a “private” kinesthetic feedback

**Figure 4:**
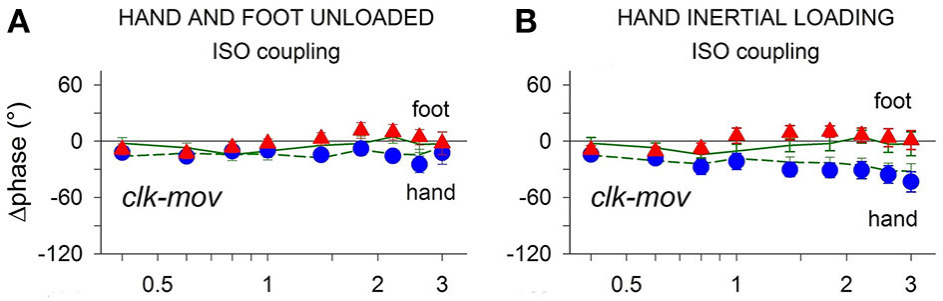
**Frequency dependent changes of the *clock-mov* phase-delay during separate oscillations of the hand and foot (green dashed and continuous lines, respectively) and during hand-foot ISO coupling (hand, blue circles; foot, red triangles)**. **(A)** When both limbs are unloaded the *clk-mov* delay of each limb, no matter whether isolated or coupled, remains nearly constant over the whole frequency range. **(B)** same relations as in **(A)** but obtained after applying an inertial load to the hand. Loading induced the expected increase of the hand *clk-mov* delay as the frequency is raised but in either limb the frequency relations obtained during separate and coupled movements still superimpose. Reproduced from Baldissera et al. (2006), © 2006 Baldissera et al; licensee BioMed Central Ltd.

As depicted in Figure 4A (Baldissera et al., 2006), the *clock-mov delay* of both the hand and the foot remains almost constant when the frequency is increased (negative values = movement delay) and is not significantly different when the limbs are moved in isolation (green solid and dashed lines) or when they are isodirectionally coupled (hand: blue circles, foot: red triangles). Enhancing the mechanical difference between the limbs, e.g., by connecting the hand to an inertial load (Figure 4B), does increase the hand phase delay with respect to the foot but is ineffective in dividing the phase curves obtained when the hand is moved alone and when it is coupled. All this holds true for antidirectional coupling too. Further details on these measurements are given in Presentation 3 in Supplementary Material.

In conclusion, the phase delay with respect to a common time-giver is apparently controlled in each limb independently from what simultaneously occurs in the other limb, even after artificially enhancing the mechanical disparity between the limbs. The control of the interlimb relative phase will thus directly result from the efficacy of the separate phase controllers of each limb.

The synchronism control of each individual limb may possibly be operated through a “private” kinesthetic feedback which provides matching of the limb movements with the motor commands linked to the central time giver. A neuro-mechanical model that faithfully simulates the operations of such a feedback (Esposti et al., 2007) is illustrated in Presentation 4 in Supplementary Material.

Added to the previously discussed evidence that the excitability changes in forearm motoneurons during foot movements are not generated by afferent signals, these last results should definitely rule out the hypothesis that the coupling coordination is achieved by a crossed kinesthetic feedback between the limbs.

## Neural mechanisms underlying the “direction principle” in hand-foot coupling

Altogether, the results reported in Section *Role of Kinesthetic Afferents from the Moving Segments in Controlling the Hand-Foot Synchronism during Coupled Movements: An Interlimb or Intralimb Feedback?* enlighten both issues presented in Section *Mechanical Differences between the Limbs and Neural Compensation for their Desynchronizing Effect on Coupled Movements*. On one side, they indicate, in a way consistent with basic physical and neurophysiological concepts, that the limbs relative phase results from an independent synchronization of each limb movement with the common clock signal. On the other side, these results also show that during foot voluntary oscillations the commands that move the foot are associated with descending subliminal commands to the “isodirectional” muscles in the resting forearm, distributed so as to facilitate ISO and contrast ANTI coupling both in the prone and supine forearm position.

Once excluded that the effects onto the resting forearm are generated by the kinesthetic afferents that monitor the foot movements, it remained to understand which physiological role these effects may have within the frame of motor control. A survey of the known motor mechanisms led to the idea that they may carry Anticipatory Postural Adjustments, APAs.

### Anticipatory postural adjustments (APAs) as possible candidates for generating the subliminal excitability modulation in forearm muscles during foot oscillations

Albeit in different conceptual and experimental contexts, the modulation of motoneuron excitability in one limb during voluntary movements of another limb had been described since several years, and explicitly discussed in many instances as due to APAs (Kasai and Komiyama, 1996; Kawanishi et al., 1999; Hiraoka, 2001).

APAs (Marsden et al., 1978, 1981; Cordo and Nashner, 1982; Bouisset and Zattara, 1987; Zattara and Bouisset, 1988; Bouisset and Do, 2008), are unconscious muscle contractions aimed at preparing fixation chains linking the segment(s) to be consciously moved to one or more firm supports, where the reaction forces to the prime movers contraction can develop (third Newton's principle) without producing any displacement. In this way it is determined which of the body segments connected by the prime movers will actually move. In the absence of a firm support, APAs will produce counter-movements that contrast the postural unbalance produced by the consciously intended movement (also said the “focal” movement). However counterintuitive it may appear, the unconscious APAs are yet a necessary and fundamental component in the generation and control of the “willed” movements.

In EMG recordings, APAs are characterized by the combined activation of muscles in one or more fixation chains, simultaneously with, or in slight advance to, the prime movers contraction. APAs scale in size with the magnitude of the primary movement (Aruin and Latash, 1996), they are influenced by the position and number of the fixation points (Slijper and Latash, 2000) and by tactile and proprioceptive information (Slijper and Latash, 2004). Also, their timing and spatial distribution may vary when the surround conditions or some feature of the movement (e.g., the direction) are changed (Cordo and Nashner, 1982; Nashner and Forssberg, 1986; Aruin and Latash, 1995). Of direct relevance in the present context are the facts that during flexion/extension of the wrist the APAs in proximal arm muscles are directionally organized (Chabran et al., 1999, 2001) and, even more interesting, that they are generated in elbow flexors when the forearm is supine but in elbow extensors when it is prone (Aoki, 1991), showing that APAs action is “isodirectional” with the primary “focal” movement whatever the forearm position.

In the past years, interest for APAs has been mainly focused on their role in stabilizing the body equilibrium, while a minor attention was devoted to their general function of providing firm support to any moving segment. For instance, the interactions between coupled limb movements and the overall body posture have been examined looking at the effects exerted by the coordinated movements on the stability of body balance (Ferry et al., 2004; Ustinova et al., 2004; Forner-Cordero et al., 2007). Here, instead, we will focus on the less investigated effects of postural anticipatory adjustments on coordination of voluntary movements (cfr. Yiou, 2005; Yiou and Schneider, 2007; Yiou et al., 2007, for the interactions between the postural dynamics elicited by arm pointing and the step initiation).

### APAs generated in forearm muscles during movements of the ipsilateral foot reproduce the same distribution pattern as the subliminal modulation

Convincing experimental evidence supports the hypothesis that the subliminal excitability changes in forearm muscles linked to the ipsilateral foot oscillation should be recognized as APAs.

When sitting on an armchair, as in the standard experiments on hand-foot coupling, body fixation is mainly obtained through the large contact surface of the posterior aspect of trunk and thighs with the seat. This would strongly attenuate the postural role of the forearm contact with its support, so that only small or subliminal APAs should develop in forearm muscles, perhaps corresponding to the excitability changes described in Section *Neural Mechanisms Underlying the “Direction Principle” in Hand-Foot Coupling*. Enhancing the forearm postural role might then transform those subliminal changes into manifest contractions.

**Figure 5:**
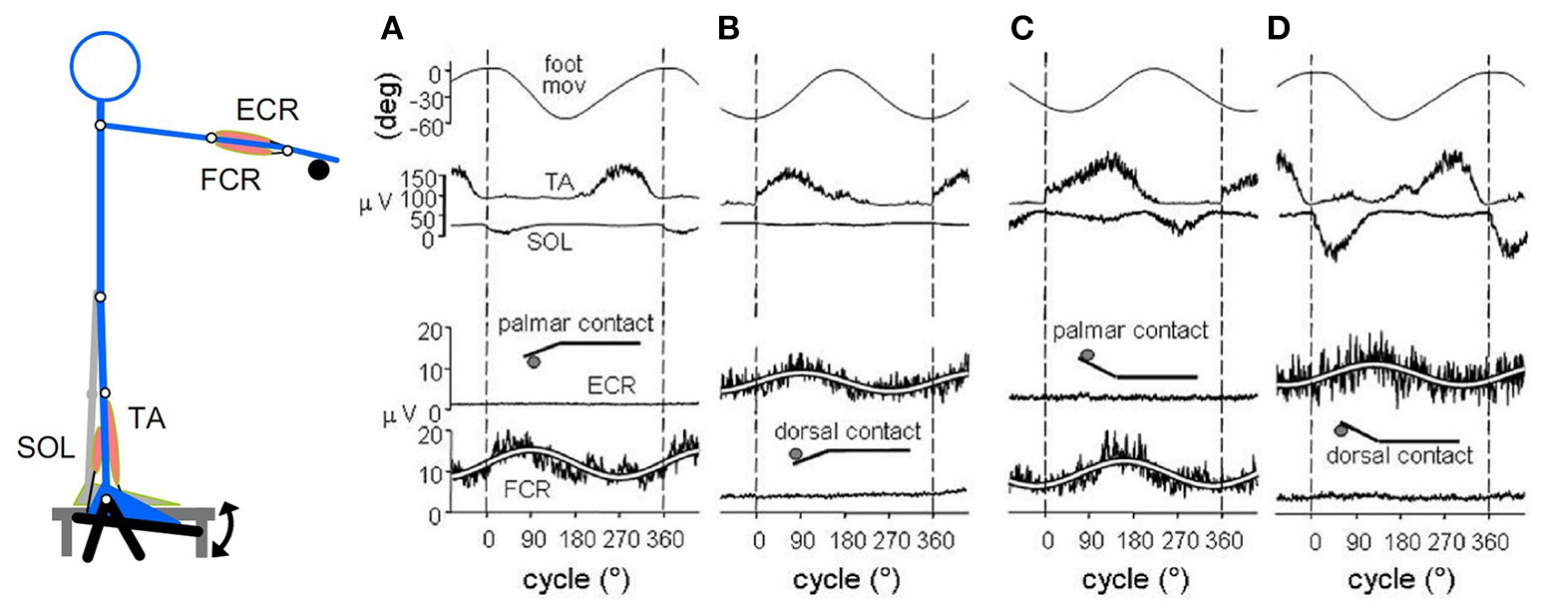
**When standing on the left foot, with the right hand touching a fixed support (inset), cyclic oscillations of right foot modulate sinusoidally the EMG activity of ipsilateral wrist muscles at the frequency of the foot movement (1.5 Hz)**. Soleus (SOL) EMG reversed. **(A)** hand prone, palmar contact; FCR EMG is cyclically modulated, the positive phase coincides with SOL activation. **(B)** dorsal contact; the positive phase of the ECR EMG modulation coincides with TA activation. **(C,D)** hand supination reverses the above pattern. In this and the following figures the white lines superimposed to the EMG recordings are the best fit sine-waves with the same period as the movements. Reproduced from Baldissera and Esposti (2005) © Wolters Kluwer Health, Inc. 2005, with permission of Wolters Kluwer Health, Inc.

Consider the following experiment. The subject stands upright, with the left foot on a stable surface, the right foot on a pivoting platform and the right arm protracted with the hand contacting a rigid support (inset of Figure 5) so as to increase the postural role of the right arm. In this asset, the cyclic flexion-extension of the right foot, which entails the risk of a forward/backward fall, is indeed counteracted by a cyclic activation of the forearm flexor and extensor motoneurons which has the same period as the foot movement (Baldissera and Esposti, 2005). If the hand is prone and in palmar contact with the support (Figure 5A), the positive phase of the modulation in FCR is coincident with the contraction of the plantar flexor SOL. Conversely, if the hand contact is dorsal (Figure 5B), activity is modulated in ECR and its positive phase coincides with the activation of TA. With the hand supine the above pattern reverses (Figures 5C,D).

**Figure 6:**
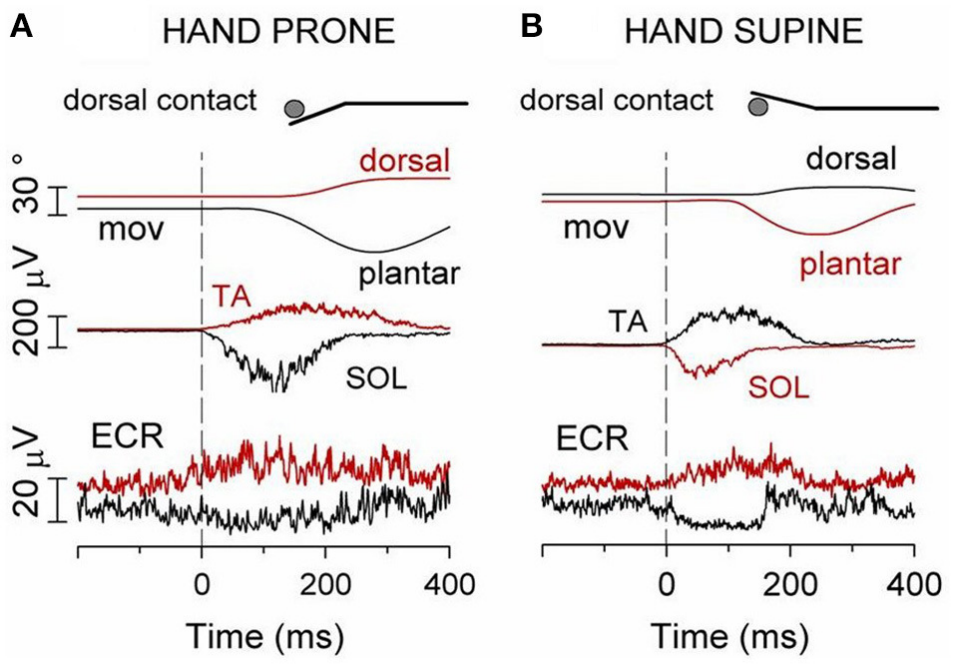
**(A)** Hand prone, dorsal contact. Red traces: TA contraction and the ensuing foot dorsal flexion are preceded by an excitatory APA in ECR, the “isodirectional” mover of the hand. Black traces: contraction of SOL and foot plantar flexion are preceded by an inhibitory APA in ECR. **(B)** Hand supine, dorsal contact. Red traces: an excitatory APA in ECR precedes contraction of the “isodirectional” SOL and foot plantar flexion. Black traces: when the movement direction is inverted to dorsal, ECR becomes “antidirectional” to SOL and the APA changes to inhibitory. Reproduced from Baldissera and Esposti (2005) © Wolters Kluwer Health, Inc. 2005, with permission of Wolters Kluwer Health, Inc.

If cyclic foot oscillations are replaced by fast flexion or extension movements, single EMG bursts fulfilling the APAs requisites develop in the “isodirectional” forearm muscles, together with a simultaneous depression of the background activity (when present) in the antagonists (Figure 6).

The strict topographical superimposition of these canonically identified APAs with the cyclic EMG activities and the subliminal excitability modulation observed when sitting would indicate that they share the same nature, even if one cannot exclude that the cyclic effects include reflex components too (see also later).

In summary, during single and cyclic foot movements and irrespective of the movement direction (plantar or dorsal flexion) and forearm position (prone or supine), excitatory burst or cyclic contractions, respectively, arise in those forearm muscles that rotate the hand isodirectionally with the foot, providing a subsidiary postural support at the hand contact. Matching these overt activities with the subliminal excitability modulation observed in the sitting position suggests that they represent two grades of one and the same event, i.e., the preparation of a fixation chain connecting the moving foot to the firm hand support. It seems therefore apparent that even when a manifest intervention of anticipatory postural adjustments is not required, subthreshold APAs may nevertheless develop, which can only be disclosed by instrumental excitability testing; and that, depending on the body postural needs, the subliminal changes can be transformed into overt APAs and viceversa.

To mechanistically explain this APAs flexibility it was imagined (Baldissera and Esposti, 2005) that any given voluntary movement is associated with the co-activation of an arborized pattern of postural commands directed toward a number of possible fixation points; and that activation of the arborization branches is supraliminal in those directed to the segments providing actual support and subliminal in those not involved at the moment in that function. Within this organization, the control of APAs might consist, from time to time, in amplifying transmission to certain targets and attenuate transmission to others, a task that could be automatically accomplished by low-level mechanisms based on afferent information.

Indeed two experimental observations support this view. On one side, studies on the feline reticulospinal neurons candidate for APA transmission (Schepens and Drew, 2004, 2006; Schepens et al., 2008) disclosed that on occasion of single forelimb movements a widespread postural activity is induced from the motor cortex in reticulospinal neurons. Such activity is then restricted to the motoneurons actually involved in postural support by selectively gating (inhibiting) at the spinal level the transmission of the APA commands to the other motoneurons, so that they remain available for voluntary activation. On the other side, in man, when two supports are available and one of the two fixation chains is voluntarily privileged, activation of the second chain is proportionally attenuated (Esposti and Baldissera, 2011) This shows that gating of the APAs commands does not take place in all-or-none fashion but is modulated in a coordinated way in the different chains.

This organization would create a pattern of subliminal activation of the motor pathways to those body segments that are not (but may potentially be) used for postural support. Even when they are subliminal, the forearm APAs described above might selectively contrast the voluntary co-activation of “antidirectional” foot and hand movers: hence the necessity of suppressing APAs by gating. In this perspective, the gating mechanism would be central in determining the hand-foot coupling preference since its attenuation or default would selectively hamper the ANTI movements.

Imagine that during flexion-extension of the right foot one needs, or wants, to simultaneously move a body segment—for instance the right hand—that belongs to one of the fixation chains that actually assist the foot movement. Following this decision, the forearm muscles would be simultaneously targeted by both the voluntary command moving the hand and the APAs command linked to the foot movement, thus being called to function as prime movers and as postural muscles at the same time.

Being dispatched to the same target, the voluntary and the APAs commands will converge at some, yet unknown, level of the motor pathways to forearm muscles. When voluntary and APA actions are isodirectional, i.e., when the postural and the voluntary commands have the same sign (either excitatory or inhibitory) this convergence will favor both actions and the previously described double goal will be met. Conversely, when the voluntary and the postural actions have opposite directions, a conflict will be generated, which may be avoided only if either the voluntary command or the APA command is suppressed. An example of voluntary movement suppression is the irresistible transition from the difficult to the easy coupling mode (Section *The “Direction Principle” in Coupling Flexion-Extension of the Ipsilateral Hand and Foot. Easy vs. Difficult Coupling Modes*). The alternative intervention, i.e., the APAs suppression aimed to free the voluntary mobilization, cannot regard a main fixation chain without challenging the steady equilibrium but it might allow voluntary activation of those muscles that actually have a minor postural function (like forearm muscles when sitting on a chair).

In conclusion, in hand-foot coupling a neural conflict between APAs and voluntary commands may occur in the motor pathways to the prime movers when the two segment are moved in opposite angular directions. The entity of the conflict should in turn depend on the APAs size, i.e, on the functional relevance of the fixation chain in which the prime movers are actually included.

## Role of APAs in differentiating ISO vs. ANTI coupling modes in other types of limb movements

In the practical impossibility of collecting from human experiments more detailed and direct evidence concerning the intimate neuronal mechanisms of the interaction between postural and voluntary actions, it seemed reasonable to search for further indirect evidence supporting the interpretation proposed for hand-foot coupling. Considering that APAs generation is intrinsic to every voluntary movement, it was decided to test whether the correlation between APAs distribution and coupling preference is also observed in other types of coupled limb movements, so as to represent a general rule of motor control.

A suitable experimental model to start exploring the reliability of this idea are the coupled movements of the arms, performed in either the horizontal (transverse) or the vertical (parasagittal) plane. Indeed, in both types of movements the moving masses and the available fixation chains remain the same, but changing the plane of arms motion would produce a topographical re-distribution of the reaction forces and moments and, consequently, of the APAs. Assuming the coupling stability to be correlated with the APAs distribution and size, it would be expected to observe stability to vary in agreement with the intervening changes in the APAs pattern.

Verifying the above idea implied measuring in the four coupling combinations, i.e., horizontal ISO (*hISO*) and ANTI (*hANTI*) and parasagittal ISO (*pISO*) and ANTI (*pANTI*), (1) the variability of the movements relative phase, SDΔΦ (i.e., the reciprocal of coupling stability, see Section *The “Direction Principle” in Coupling Flexion-Extension of the Ipsilateral Hand and Foot. Easy vs. Difficult Coupling Modes*), to be matched, in each coupling combination, with (2) the distribution of the postural adjustments associated with unidirectional and cyclic arm movements.

### Coupling coordination of ISO vs. ANTI cyclic movements of the arms in the horizontal and parasagittal planes

A directional easy-vs.*-*difficult polarization is found in arm coupled movements performed in either plane of motion, being however opposite in horizontal movements (ANTI vs. ISO) with respect to parasagittal movements (ISO vs. ANTI) (Baldissera et al., 2008b; Baldissera and Esposti, 2013). Indeed SDΔΦ is lower (i.e., coupling stability is higher) in *hANTI* and *pISO* than in both *hISO* and *pANTI*. No significant SDΔΦ difference is found between the two easy modes while SDΔΦ is higher in *hISO* than in *pANTI*. Consequently, the stability loss between easy and difficult coupling is larger in horizontal than in parasagittal movements. Summing up, the instability (difficulty) of the four movement combinations, as evaluated by SDΔΦ, increases along the scale *hANTI* ≠ *pISO* < *pANTI* < *hISO*.

### APAs associated with arm movements in the horizontal and parasagittal plane

**Figure 7:**
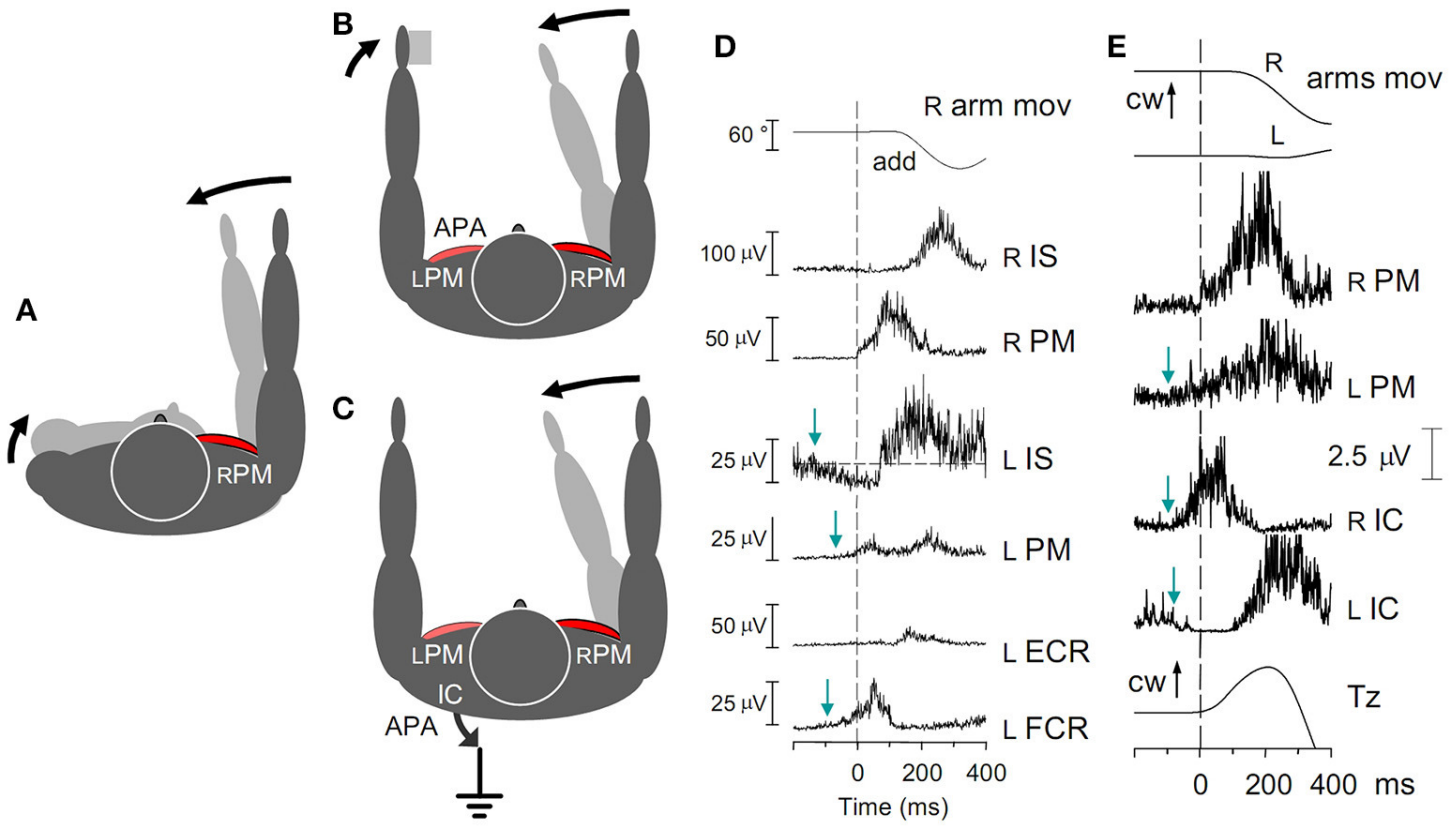
**(A)** Contraction of the right rPM in the absence of any fixation chain would counter-rotate the right arm and the trunk. A pure arm movement is obtained if the trunk rotation is opposed by APAs in the fixation chain between the arms **(B)** and/or to the ground **(C)**. **(D)** Fast adduction of the right arm in the horizontal plane, prime mover right rPM. Subject sitting on a turnable chair, palmar surface of the left hand touching a fixed support. Excitatory APAs develop in left arm muscles lPM and lFCR, inhibitory APAs in lIS. All APAs precede the prime mover contraction (dashed line). No inhibition is seen in lECR, due to the absence of any background activity. **(E)** Activation of the rPM is preceded by excitatory APAs in both the lPM and the rIC, while lIC is simultaneously inhibited. The related change of Tz starts simultaneously with the rPM activation. cw, clock-wise. Reproduced from Baldissera et al. (2008a,b), © Springer-Verlag Berlin Heidelberg 2008, with permission of Springer.

The reaction forces to movements of one arm in either the horizontal or parasagittal planes, are discharged by two main fixation chains, to the contralateral arm and to the ground, respectively (cfr. Figure 7 for horizontal movements).

The relative engagement of each chain varies according to the body asset. In the standing position, the major anticipatory postural activation usually develops in muscles of the chain to the ground, but when the ground surface is slippery (e.g., iced), or when sitting on a turnable chair, the role of APAs in muscles of the contralateral arm becomes relevant.

Distribution of the muscular and mechanical APAs linked to unidirectional movements of one and, more rarely, both arms was already reported by several authors (e.g., Belen'kii et al., 1967; Friedli et al., 1984; Bouisset and Zattara, 1987; Zattara and Bouisset, 1988; Aruin and Latash, 1995; Hodges et al., 1999, 2000; Shiratori and Aruin, 2004; Bouisset and Do, 2008; Tomita et al., 2010). In the perspective discussed here, however, these data had to be supplemented with some further observation.

#### APAs in arm adduction-abduction in the horizontal plane

##### Fast unidirectional movements of the right arm

When standing upright, the voluntary adduction of the right arm in the horizontal plane (prime mover right *Pectoralis Major*, rPM) exerts at the shoulder a torque that tends to rotate the trunk clockwise (Figure 7A). This torque is contrasted by the body inertia and actively counteracted by a counterclockwise torque simultaneously generated by the two fixation chains mentioned above (Figures 7B,C), in different proportions in the various contexts (Baldissera et al., 2008a,b).

*Fixation chain between the arms*. During a fast adduction of the right arm, if a rigid support is available to the left hand (Figure 7B) APAs develop in the homologous left-side adductor, lPM, and in the wrist flexor lFCR (Figure 7D); meanwhile in the left *Infraspinatus*, lIS, the background EMG activity (if present) is reciprocally depressed. These APAs discharge the primary rotational perturbation onto the support and arrest the clockwise rotation of the trunk. When no support is available, APAs in lPM fixate the left arm to the trunk, thus increasing the overall inertia, or even produce an adduction of the left arm and a mirror reaction torque, in either case attenuating the trunk rotation.

*Fixation chain to the ground*. A counterclockwise torque is also exerted on the thorax by APAs in the trunk and lower limb muscles (Figure 7C), an example of which are the prominent burst in the right *Ischiocruralis* muscle, rIC, and the simultaneous anticipatory depression of lIC (Figure 7E). These asymmetric APAs generate at the ground a clockwise reaction torque about the vertical axis, *Tz*, in the direction opposite to the arm acceleration.

**Figure 8:**
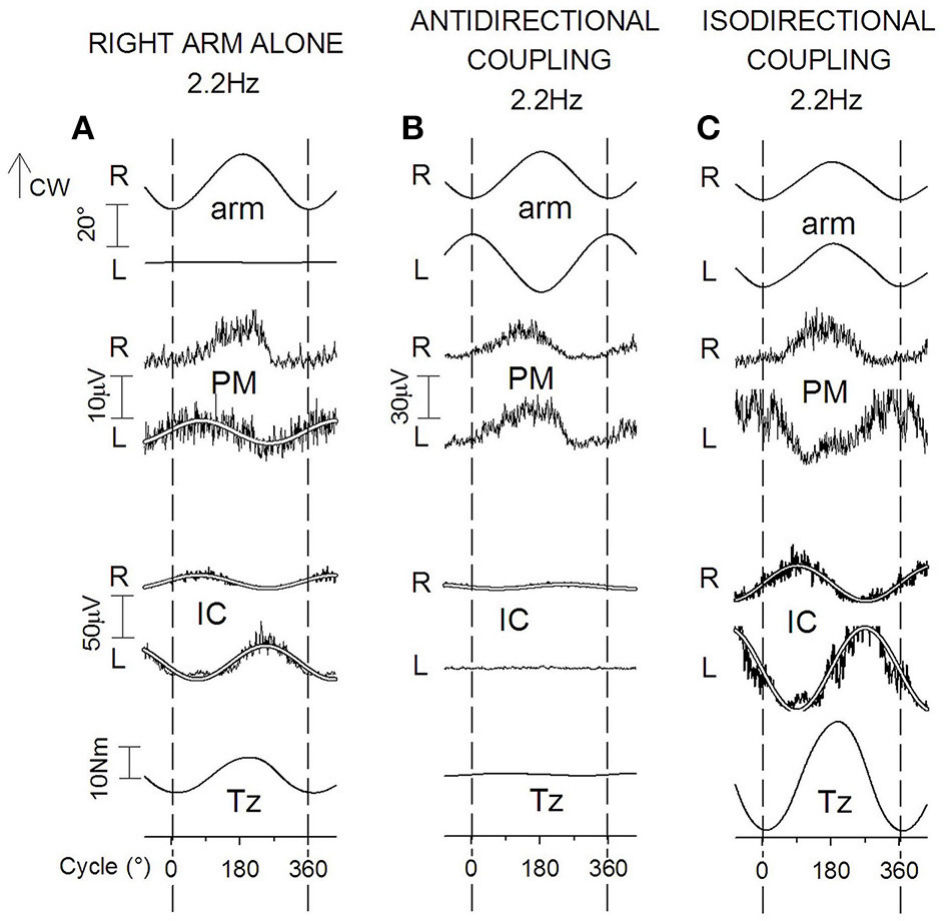
**Cyclic postural adjustments during rhythmic oscillations in the horizontal plane of the right arm alone (A)** and of both arms in ANTI and ISO coupling **(B,C)**. Prime movers, rPM in **(A)**, rPM and lPM in **(B,C)**. During one arm movements, cyclic APAs develop both in lPM and, phase-opposite, in rIC and lIC. This same pattern of EMG modulation, but doubled in size, is present in IC muscles during ISO (difficult) coupling **(C)**. During ANTI coupling, instead, activity is absent in lIC and minimal in rIC **(B)**. In all coupling modes, *Tz* undergoes changes parallel to the IC activities; cw, clock-wise. Reproduced from Baldissera et al. (2008b), © Springer-Verlag Berlin Heidelberg 2008, with permission of Springer.

When the right arm is cyclically oscillated, in both fixation chains the above APAs are replaced (Figure 8A) by a sinusoidal EMG modulation with the same period as the right arm oscillations (Baldissera et al., 2008b). In both arm (lPM) and leg (rIC) muscles, the modulation phase-advances the rPM cyclic activity, its absolute timing being quantitatively congruent with the time-anticipation of the APAs linked to unidirectional movements.

##### Cyclic coupled adduction-abduction of both arms

During coupled arm movements, APAs in arms muscles cannot be distinguished from the voluntary activation, and only APAs in the fixation chain to the ground can be analyzed.

When cyclic movements are mirror symmetrical (ANTI), EMG modulation in lIC and rIC is absent or marginal over the whole frequency range (1.0–3.0 Hz), justifying the virtual annihilation of *Tz* (Figure 8B). Conversely, during arms ISO movements (Figure 8C), the amplitude of the phase-opposite modulation of rIC and lIC EMG and the size of *Tz* are much larger than in one-arm movements and this difference steeply increases when the frequency is raised (see also Section *Effects of APAs in the Fixation Chain to the Ground on Coupling Coordination of Horizontal and Parasagittal Arm Movements*).

The view that the APAs and the sinusoidal EMG modulation share the same nature is not only suggested by the identical distribution and the temporal compatibility of the two events, but it is also supported by the observation (Leonard et al., 2011) that when the target of a pointing task is unexpectedly shifted after the movement had started, the correction movement is preceded by legs postural adjustments in the same way as at the movement initiation, showing that the online correction of voluntary finalized movements includes both the conscious and the associated APA commands. If one considers that cyclic movements are continuously changing in velocity and direction so as to require uninterrupted control of trajectory and timing, it is reasonable that the related APA commands are continuously updated over the entire sinusoidal course of movements. Thus, although the possibility exists that these cyclic postural activities may include reflex components too, we will continue hereafter to name the cyclic postural activities as APAs.

#### APAs in arm flexion-extension in the parasagittal plane

##### Fast unidirectional flexion or extension

The reaction forces to arm movements in the parasagittal plane are discharged by APAs in the same two fixation chains as horizontal movements, but with a different topographical distribution. For a mechanical analysis of these effects, see Esposti and Baldissera (2013).

##### APAs in the fixation chain between the arms

**Figure 9:**
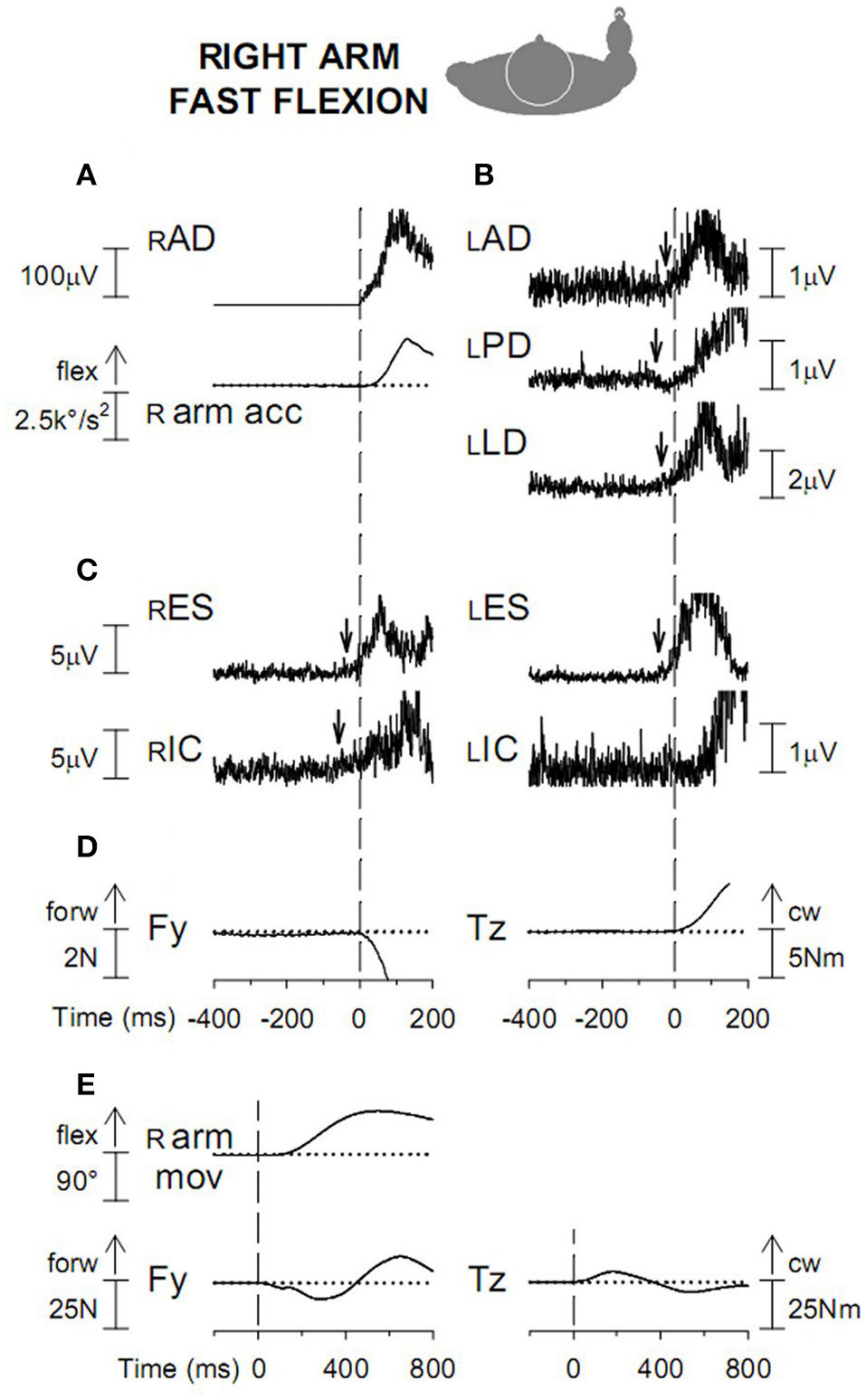
**(A)** Voluntary activation of rAD and acceleration (r arm acc) of the ensuing arm flexion. Vertical dashed line: onset of the rAD EMG burst. **(B)** APAs in the left arm muscles, excitatory in lAD and lLD and inhibitory in lPD. **(C)** excitatory APAs in trunk muscles rES and lES and in the right leg muscle rIC. **(D)** anterior-posterior force *Fy* and clockwise moment about the body vertical axis *Tz*, discharged to the ground. **(E)**. *Fy* and *Tz* changes are displayed on a longer time base. Dashed line: onset of prime mover burst; cw, clock-wise. Reproduced from Esposti and Baldissera (2013), © Springer-Verlag Berlin Heidelberg 2013, with permission of Springer.

Supplementing the scanty data available in the literature (Zattara and Bouisset, 1988) it was disclosed (Esposti and Baldissera, 2013) that a fast flexion (Figures 9A,B) or extension of the right arm (prime movers right *Anterior Deltoid*, rAD, and *Posterior Deltoid* rPD, respectively) elicits APAs in the left side homologous lAD and lPD so as to replicate the excitation and inhibition pattern of rAD and rPD. In the left extensor *Latissimus Dorsi*, lLD, APAs are instead opposite to the voluntary actions in rLD.

##### APAs in the fixation chain to the ground

Many details of the APAs in trunk and legs associated with flexion-extension of one or both arms had been previously reported (see above for references, cfr. also Zattara and Bouisset, 1988; Shiratori and Aruin, 2004; Bleuse et al., 2005; Bouisset and Do, 2008; Morris et al., 2013) and some were confirmed. Fast flexion of the right arm (Figure 9C) is associated with symmetric APAs in the right and left *Erector Spinae* (rES and lES) and asymmetric APAs in rIC and lIC, excitatory on the right side during arm flexion (cfr. Zattara and Bouisset, 1988; Bleuse et al., 2005) and on the left side during extension. An anticipatory change of the anterior-posterior force *Fy* and of the torque *Tz* are meanwhile discharged to the ground (Figures 9D,E). During single arm oscillations all the above APAs are replaced by EMG modulation cycles with the same period as the arm movement and increasing in size when the movement frequency is raised (Baldissera and Esposti, 2013).

##### Cyclic coupled flexion-extension of both arms

**Figure 10:**
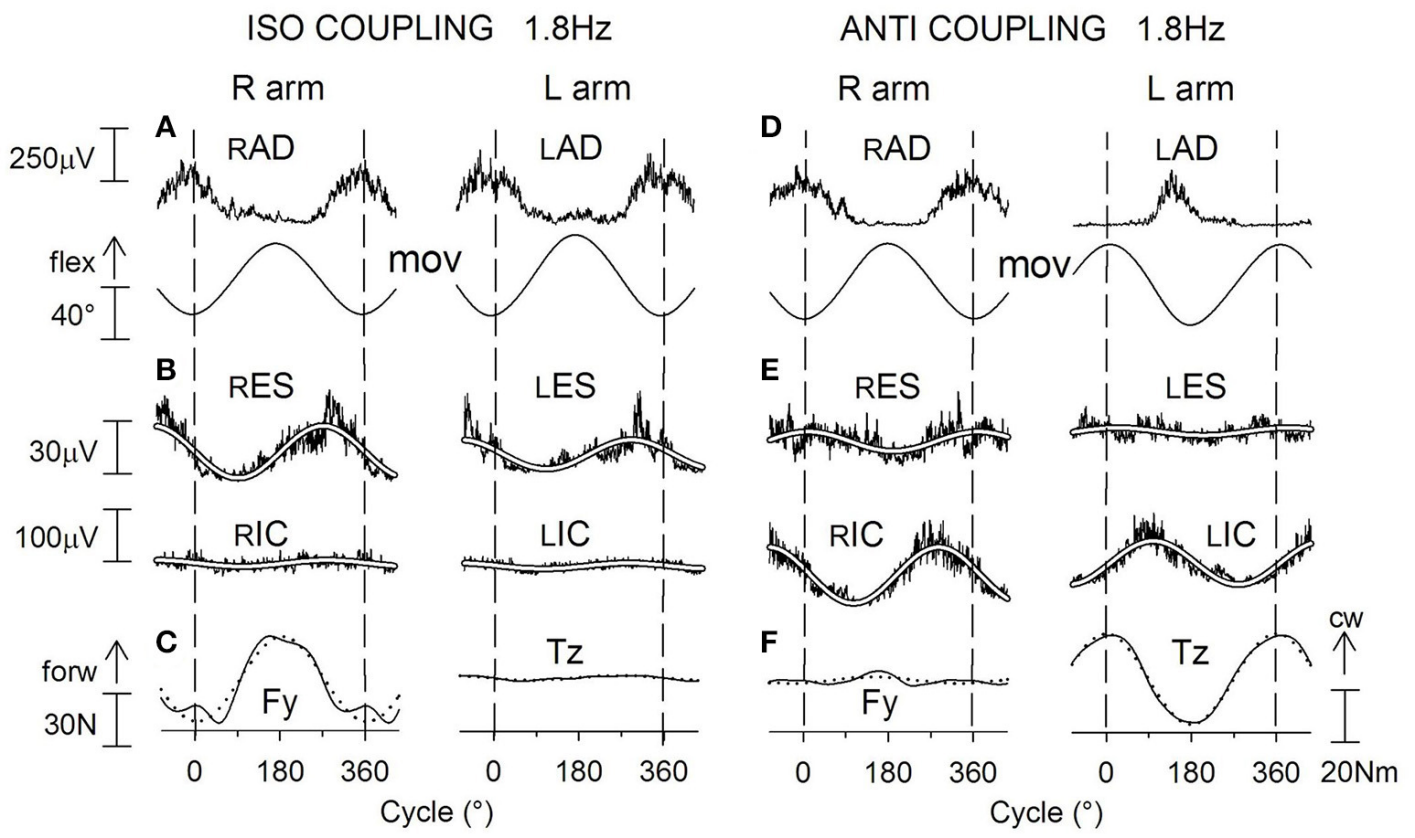
**Voluntary oscillations of both arms in the parasagittal plane**. ISO and ANTI coupling. Same labels as in Figure 9. **(A,D)**: cyclic voluntary EMG activity in rAD and lAD muscles and ensuing oscillations of the right and left arms. In ISO coupling the activation of both trunk (ES) and thigh (IC) postural muscles is synchronous on the two sides **(B)** and corresponds **(C)** to a clear-cut anterior-posterior ground reaction *Fy* while the torque *Tz* is marginal. **(E)** in ANTI coupling, the EMG modulation in rES and lES remains synchronous but consistently lower with respect to ISO, whereas the IC activation is increased in size and is phase-opposite in rIC and lIC. Correspondingly **(F)**, a large reaction torque *Tz* is generated while *Fy* is strongly reduced. Reproduced from Baldissera and Esposti (2013), © Springer-Verlag Berlin Heidelberg 2013, with permission of Springer.

During ISO arms oscillations (Figure 10A) cyclic APAs develop symmetrically both in rES and lES and in rIC and lIC (Figure 10B), as requested to cope with the anterior-posterior perturbation. When frequency is raised, APAs in both muscles pairs increase in size remaining synchronous on the two sides. The anterior-posterior reaction force *Fy* undergoes a sinusoidal modulation with the same period as the arm movements (Figure 10C) and significantly increases in size when the frequency increases. Conversely, the size of *Tz* remains negligible throughout the whole frequency range.

During ANTI coupling (Figures 10D–F) the APAs modulation in rES and lES is significantly smaller than in ISO, but it increases in either mode as the frequency is raised. The phase difference between the two sides is highly variable among subjects and, on the average, half-way between 0° and −180°. Cyclic APAs activation is phase-opposite in rIC and lIC and, when the frequency is raised, it increases in size to a greater extent than in ISO. Concerning the ground reactions, the sinusoidal modulation of *Fy* remains very low over all the frequency range, while the torque *Tz* significantly increases between 1.0 and 2.2 Hz but progressively decreases by about the same amount between 2.2 and 3.4 Hz (see later). The phase of *Tz* oscillations during ANTI is opposite to that of the right arm movements and is not significantly affected by frequency.

In summary, the coupled arm oscillations in the horizontal and parasagittal plane are distinguished because the preferred (more stable) coupling mode is ANTI in horizontal and ISO in parasagittal movements. Both movement types, however, share the common feature that the unstable coupling mode is the one in which: (1) in the motor pathways to the prime movers of each arm the voluntary commands have opposite sign with respect of the APAs commands linked to the voluntary activation of the contralateral arm (cfr. Section *APAs Associated with Arm Movements in the Horizontal and Parasagittal Plane*); and, (2) the prime movers generate torsional perturbations, which are discharged by APAs to the ground by producing a reactive torque *Tz*.

### Interactions betweeen APAs and voluntary commands during coupled arm movements

Combining the results presented in Sections *Coupling Coordination of ISO vs. ANTI Cyclic Movements of the Arms in the Horizontal and Parasagittal Planes and APAs Associated with Arm Movements in the Horizontal and Parasagittal Plane* suggests that the selective constraints affecting the two difficult modes, *hISO* and *pANTI*, may be related to the APAs that occur in both the fixation chains, between the arms and to the ground.

In the following, the two mechanisms will be discussed separately.

#### Neural interference between APAs and voluntary commands in the motor pathways to the prime movers in horizontal and parasagittal arm movements

##### Horizontal movements

During ANTI (mirror symmetrical) coupling, voluntary, and postural commands to the prime movers have the same sign (i.e., both excitatory or inhibitory). Accordingly, they should potentiate each other. However, it should be noted that in ANTI coupling the two arms exert on the trunk opposite torques that physically cancel each other, so that no postural adjustment is needed. Indeed, no APAs are generated during ANTI in the fixation chain to the ground (Figure 8B) and, reasonably, APAs should be absent in the contralateral arm too, thus leaving movement coupling unaffected.

During ISO coupling the APAs elicited in the prime movers of either arm are opposite in sign with respect to the voluntary commands. Hence, maintaining the movement amplitude, as done in the experiments described here, would require that either the voluntary commands are increased or APAs are actively suppressed (gated). The former intervention, however, would be doomed to fail since when the frequency (velocity) of the arm movements increases, the size of APAs also increases (Baldissera et al., 2008b, see also Shiratori and Aruin, 2007).

##### Parasagittal arm movements

The interaction between voluntary and APAs commands is somewhat more complex in parasagittal than in horizontal oscillations (cfr. discussion in Baldissera and Esposti, 2013).

In the motor pathways to *Anterior* and *Posterior Deltoid*, the voluntary commands and the APAs linked to the movement of the contralateral arm have the same sign during ISO, thus facilitating or leaving unaffected (see above) the coupled movements. Conversely, during ANTI coupling, voluntary commands and APAs have opposite sign and maintaining the movement amplitude requires APAs to be actively suppressed (gated).

In the motor pathways to *Latissimus Dorsi*, instead, APAs and voluntary commands have opposite sign during ISO (see Section *APAs in Arm Flexion-Extension in the Parasagittal Plane*), thus creating a local neural conflict during the preferred mode.

##### Escape from APAs suppression

As discussed in Section *Anticipatory Postural Adjustments (APAs) as Possible Candidates for Generating the Subliminal Excitability Modulation in Forearm Muscles during Foot Oscillations for hand-foot coupling*, the conflict arising in both horizontal and parasagittal movements when APAs and voluntary commands have opposite signs can be solved with the intervention of the control mechanism that provides gating of transmission of the APAs commands (Schepens and Drew, 2004, 2006; Schepens et al., 2008). One sign of such intervention may be the higher increase of cerebral activation during non-preferential coupling compared to preferential coupling (Sadato et al., 1997; Debaere et al., 2001, 2004; Immisch et al., 2001; Ullen et al., 2003).

However, as the oscillation frequency increases, the size of APAs also increases, implying that the gating mechanism for APAs suppression must be intensified in parallel. Descriptively, the decay in coupling stability observed when either the movement frequency is raised or the exercise duration is prolonged, fits the general description of fatiguing systems. It may therefore be supposed that during “difficult” coupling the gating system undergoes neural “fatigue” (linked to the turnover of synaptic transmitters, adaptation, or potentiation phenomena, etc.) proportionate to the frequency and duration of the exercise, so as to progressively attenuate the suppression of APAs transmission. This, in turn, would increase the coupling instability and favor the transition to the opposite coupling mode.

#### Effects of APAs in the fixation chain to the ground on coupling coordination of horizontal and parasagittal arm movements

The major clue suggesting that in both movement types APAs in the chain to the ground may exert an indirect influence on coupling coordination is the increase of the chained postural actions observed when passing from the more stable to the less stable coupling mode (Sections *APAs in Arm Adduction-Abduction in the Horizontal Plane and APAs in Arm Flexion-Extension in the Parasagittal Plane*). Evidence for such an effect has also been reported by Van der Woude et al. (2008) for synchronous and alternate hand cycling. Aimed to better elucidate this correlation, the effort sustained by the postural chain to the ground was tentatively quantified (1) by the forces discharged to the ground and (2) by the energy cost of the chain actions.

##### Ground reactions Fy and Tz

Each of the EMG and mechanical variables pertaining to the fixation chain to the ground is exclusively related to either *Fy* or *Tz* (Esposti et al., 2013). The latter can then be taken as indicators of the overall entity of postural actions occurring in the chain.

**Figure 11:**
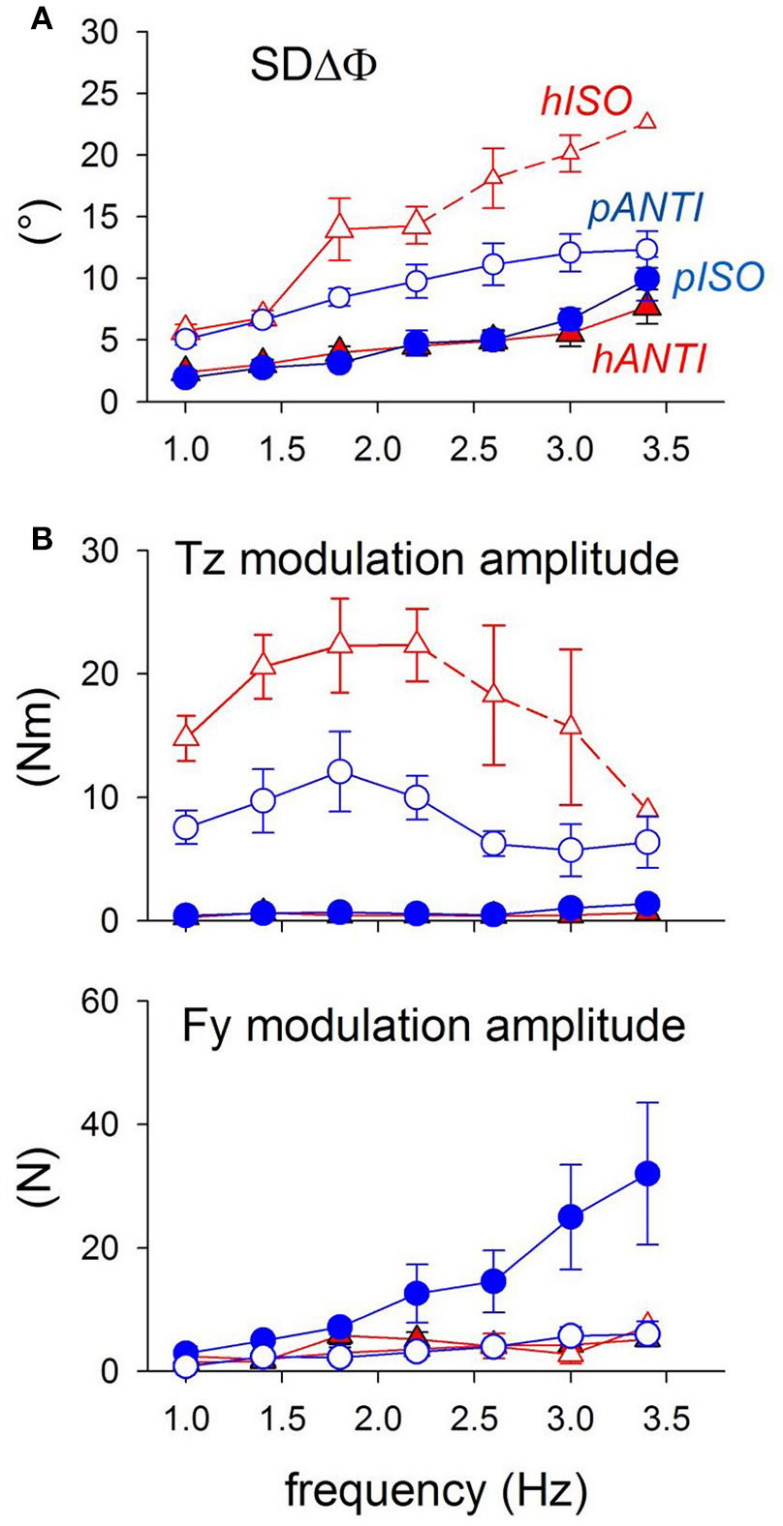
**Coordination marker SDΔΦ and postural markers *Fy* and *Tz* recorded during *c*oupled arm movements performed by the same subjects in both the horizontal and parasagittal planes (red triangles and blue circles, respectively)**. Filled symbols, stable (easy) modes; open symbols, unstable (difficult) modes. **(A)** SDΔφ is identical in the easy modes *pISO* and *hANTI*, larger in *pANTI* and even more so in *hISO*. Small symbols and dashed lines: values obtained in only part of the subjects. **(B)** *Tz* is virtually null in the two easy modes (filled symbols) and not significantly different between the two movement types. Instead, it increases with frequency in the two difficult modes (open symbols), more in *hISO* than in *pANTI*. Above 2.0–2.4 Hz, *Tz* starts however to decrease progressively. Size of *Fy* increases monotonically with frequency up to 3.4 Hz in *pISO* while it remains negligible in the other three movement combination. Reproduced from Baldissera and Esposti (2013), © Springer-Verlag Berlin Heidelberg 2013, with permission of Springer.

In a group of subjects performing both horizontal and parasagittal movements in the same experimental session, no significant *Tz* changes are observed (Figure 11B) at any frequency in the two more stable “easy” modes (*pISO* and *hANTI*). Conversely, *Tz* is large and increases with frequency in both the “difficult” modes, significantly more in *hISO* than in *pANTI*. Thus, the distribution gradient for *Tz* among the four movements combinations is: *hANTI* ≠ *pISO* < *pANTI* < *hISO*, i.e., just the same as for SDΔΦ.

The anterior-posterior force *Fy* is appreciable and increases significantly with frequency only during ISO parasagittal movements (Figure 11B) thus appearing as unsuitable for discriminating among the four movement conditions.

Note, however, that in both movement types *Tz* decreases above 2.0–2.5 Hz while SDΔΦ continues to grow (compare Figure 11A and Figure 11B). This discrepancy might disprove the existence of a causal link between postural effort and coupling stability but it may also indicate that in the high frequency range *Tz* does not express all the forces generated for postural aims because part of them are “absorbed” within the different chained segments of the trunk by opposite muscular actions.

##### Oxygen uptake during coupling of horizontal and parasagittal movements

Theoretically, in both horizontal or parasagittal movements the mechanical work for voluntarily oscillating the two arms together should be equal in ISO and in ANTI, insofar as the movements amplitude and frequency are identical in the two modes. Since these two last conditions were respected in the present experiments, the muscle force and the metabolic consumption for moving the arms should be the same in ISO and ANTI modes. If so, for each movement type, the difference in energy consumption between the stable and unstable coupling modes should represent the cost of the global postural effort in the fixation chain to the ground.

The energy cost of each exercise, Δ$\dot{\text{V}}$O_2_ (i.e., the difference between the steady-state oxygen uptake at rest and during the exercise) was evaluated in two groups of subjects during arm oscillations in the horizontal and parasagittal plane, respectively, and normalized for both the body mass and movements amplitude (Esposti et al., 2010, 2013).

**Figure 12:**
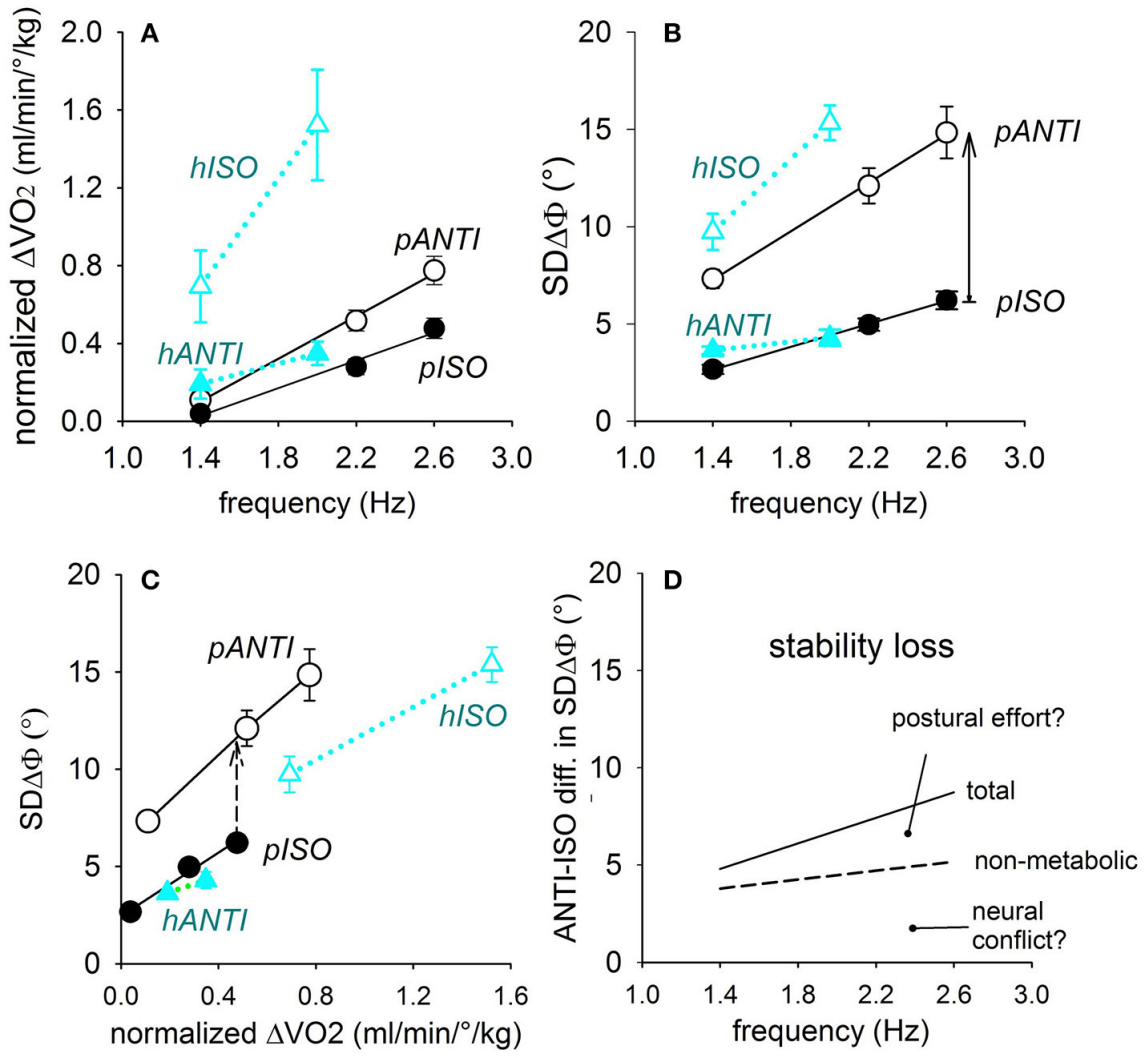
**Relations of the oscillation frequency with the normalized oxygen uptake Δ$\dot{\text{V}}$O_2_ (A)** as well as with the coupling variability SDΔΦ **(B)**, in either coupling mode of the horizontal and parasagittal movements (black and blue symbols, respectively). In **(C)**, direct correlation between SDΔΦ and Δ$\dot{\text{V}}$O_2_. The SDΔΦ-Δ$\dot{\text{V}}$O_2_ relation in the two difficult modes (*pANTI* and *hISO*) runs higher and has a higher slope than in the respective easy modes (*pISO* and *hANTI*). Continuous arrow in **(B)**: total ISO-vs.-ANTI stability loss; dashed arrow in **(C)**: stability loss independent from Δ$\dot{\text{V}}$O_2_. Both are marked at 2.6 Hz. In **(D)** the total and the non-metabolic stability losses in parasagittal movements are plotted by a continuous and dashed lines, respectively, so as to separate the two components of the stability loss, namely the *neural conflict* and the *postural effort*. Reproduced from Esposti et al. (2013), © Springer-Verlag Berlin Heidelberg 2013, with permission of Springer.

In *horizontal movements* (Figure 12A, blue triangles) Δ$\dot{\text{V}}$O_2_ is quite larger in *hISO* (difficult) than *hANTI* (easy) and increases in both modes when the frequency is raised from 1.4 to 2.0 Hz, to a larger extent in ISO than in ANTI. The related values of SDΔΦ are distributed following the same pattern (Figure 12B).

In *parasagittal movements* (Figure 12A, black circles), Δ$\dot{\text{V}}$O_2_ is higher in *pANTI* (difficult) than in *pISO* (easy) and shows a highly significant linear correlation with frequency in both coupling modes, with a higher slope in ANTI than in ISO. The corresponding changes of SDΔΦ also show a highly significant linear correlation with frequency (Figure 12B).

In conclusion, the normalized Δ$\dot{\text{V}}$O_2_ varies across the four movement combinations along the gradient: *hANTI* ≠ *pISO* > *pANTI* > *hISO*, analogously to the gradient for SDΔΦ measured in these and previous experiments.

Figure 12C relates SDΔΦ with Δ$\dot{\text{V}}$O_2_ and shows that in *parasagittal movements* at any given level of Δ$\dot{\text{V}}$O_2_, SDΔΦ is definitively higher (i.e., coordination is worse) in *pANTI* than in *pISO*, signaling that a quote of the ISO vs. ANTI stability loss is not related to a change in oxygen uptake, (i.e., to the postural effort), but to some non-metabolic factor(s), presumably to the neural conflict between APAs and voluntary commands.

The metabolic and non-metabolic quotes can be separated from each other. On one side, the quote of the stability loss independent from the oxygen uptake is calculated by subtracting from each other the ISO and ANTI linear relations in plot Figure 12C. On the other side, subtraction of the ISO and ANTI linear relations in plot Figure 12B measures the total stability loss, due to both the metabolic and the non-metabolic components. The total and the non-metabolic losses are shown in plot Figure 12D (continuous and dashed lines, respectively) over the range of the frequencies tested, allowing to identify the quote of the stability loss linked to the increase of Δ$\dot{\text{V}}$O_2_, i.e., to the postural effort of the fixation chain to the ground.

##### Mechanism for the coupling destabilizing effect of the postural effort

Hypothesizing a causal relationships between SDΔΦ and the oxygen uptake is possibly supported by the repeated observation that fatigue of either postural or focal muscles modifies APAs timing and size (Morris and Allison, 2006; Strang and Berg, 2007; Kanekar et al., 2008; Strang et al., 2008, 2009). Even if the fatigue levels attained in the cited experiments were not reached in the present exercises, one might argue that the intensified recruitment of both the prime and the postural muscles of trunk and legs during the non-preferred coupling may tend to alter the phase relationships of APAs with respect to the prime movers activity. A control mechanism for maintaining the synchronization between the voluntary and postural components of the movements should then be required, which would progressively become more expensive as the movement frequency increases, giving rise to fatigue phenomena similar to those proposed for the decay of APAs gating in the motor pathways to arm muscles (Baldissera et al., 2008b; Baldissera and Esposti, 2013). If this were to be the case, the stability loss in the less-coordinated coupling mode may result from a unique phenomenon, i.e., the exhaustion of the control mechanisms that govern the interferences arising at different neuronal levels between postural and conscious voluntary commands.

#### Flexion-extension movements of the hand in the horizontal plane

In a group of subjects whose horizontal movements of the arms had been previously analyzed, the same experimental scheme was followed to investigate coupled flexion-extension movements of the hands in the horizontal plane (Baldissera et al., 2008b). As for arm horizontal movements, SDΔΦ of the hand oscillations is significantly higher (i.e., coupling stability is lower) in ISO than in ANTI coupling, in conformity with the APAs distribution in the two modes.

### Rules of interlimb coupling: a special case of APAs physiology

The ensemble of the above results demonstrates that the easiness/difficulty of coupled limb movements does not depend on the associated muscles, neither on the ISO or ANTI modality *per se*, nor on the limbs being ipsilateral or symmetric. It strongly suggests, instead, that the coupling preference depends, for each type of coupled movements, on the distribution and size of the anticipatory postural adjustments that assist those movements. While performing their fundamental function of body fixation and stabilization during voluntary movements, APAs fatally generate some undesired “side effect” too. This happens when, during a certain focal movement, one simultaneously wants to move another body segment, belonging to a fixation chain, in the direction opposite to the APAs actions on that same segment. If a main chain reaching the ground is involved, where APAs are strong, performing the voluntary movement would require the APAs to be suppressed (gated) but, in this way, the static equilibrium would be lost. If instead the chain is a secondary one, APAs can be reduced via gating to a weak or subliminal level so that coupling of the two movements becomes possible while the movements are slow. Increasing the movement frequency, then coupling becomes unstable, difficult or even impossible, supposedly due to the exhaustion of the gating mechanism (Section *Neural Interference between APAs and Voluntary Commands in the Motor Pathways to the Prime Movers in Horizontal and Parasagittal Arm Movements*) and to the increased postural effort (Section *Effects of APAs in the Fixation Chain to the Ground on Coupling Coordination of Horizontal and Parasagittal Arm Movements*).

Since APAs are excitatory or inhibitory depending on the direction (in extrinsic coordinates) of the focal movement, this would explain why a “direction principle” rules the dichotomy of easy vs. difficult coupling.

Finally, it has to be expected that any factor that modifies the generation, execution, and organization of the APAs (for instance factors depending on the individual variability, on changes in the sensory context or in the body attitude, on training or on pathological events, see Section *Postural Constraints from Neuroscience to Sports and Rehabilitation Medicine*), will affect the coordination of limb coupled movements too. It cannot of course be excluded that other yet unknown factors may add to the APAs in destabilizing limbs coupling.

## Postural constraints from neuroscience to sports and rehabilitation medicine

In the above Chapters some of the rules of the anticipatory postural control were elucidated, with special reference to the APAs interference with interlimb coordination. Which is the potential translation of this knowledge beyond theoretical neuroscience?

### “Will” develops by entangling focal and postural movements

APA control is an intrinsic component of voluntary movement, not an “external” phenomenon: by definition, APAs exist as far as a movement is voluntary. Yet, APA develop quite independently and much later with respect to the capacity to imagine a voluntary movement. APA control is only primitively organized in the newborn (see Girolami et al., 2010 for references), consistently with the immature myelination of the central nervous system at birth. Once their neurobiological substrate is mature, focal, and postural movements remain entangled during further motor learning.

The APA component of learning is more relevant the more the movement involves long chains of muscles for within-body and body-ground stabilization.

#### APA as a candidate ”neural factor” in force development and loss

The APA physiology provides a promising model for the explanation of the well-known, yet not entirely understood, “neural” mechanisms underlying the effect of resistance/power training. “Resistance” training, aimed at improving force and power in voluntary movements through muscle hypertrophy, is most commonly based on exercises of heavy lifting efforts which, despite high risk to destabilize the body system, are usually perceived as rather simple to learn and perform. Yet, unspecified “neural” factors (Sale, 1988) have long been postulated to explain (a) why force gains anticipate (even by many weeks) muscle hypertrophy (Clark et al., 2008) and (b) why, although the focal main muscles may stay the same in different exercises, the force gain is higher for the trained and very similar movements (Wirth et al., 2016). Consistently enough, force training with fast movements entails the highest force gain in movements performed at the trained speed; unilateral training allows higher unilateral force gains, compared to bilateral training, etc. (Jones and Rutherford, 1987). Unspecified “neural” factors, supported by TMS (Pearce et al., 2013) and fMRI (Farthing et al., 2011) findings, have also been claimed to subtend cross-education, i.e., force gains on the same movement of the untrained side, or force maintenance after immobilization, thanks to force training of the opposite side, as well as force gains achievable after pure mental practice (Yue and Cole, 1992; Reiser et al., 2011). In all cases, results may depend on the specific postural chains engaged, and thus unconsciously trained or not, in different exercises. De-training leads to force loss higher than the loss in muscle mass (Narici et al., 1989).

#### APA as a candidate ingredient of skill acquisition and loss and motor learning

Recent studies evidenced that APA efficiency actually subtends also skill, and not only force, of voluntary movements. For instance, earlier APAs in shoulder muscles have been shown to be associated with higher accuracy in pointing tasks (Caronni et al., 2013), and in the greater accuracy of pointing with the dominant, compared to the non-dominant upper limb (Bruttini et al., 2016).

De-training also conceals a loss of APAs efficiency. After only a 12 h wrist and fingers immobilization a simple finger tapping task is altered, due to insufficient stabilization of the elbow, reflecting delayed activation or inhibition of proximal muscles (Bolzoni et al., 2012). In general, an improvement of APAs has long been suspected as a potential component of learning of voluntary movements (Rutherford and Jones, 1986).

Yet the relationships between APAs and learning were not extensively investigated in the subsequent literature. It was shown, however, that exercises requiring high motor skills rapidly lead to improvements in balance (Aruin et al., 2015; Kanekar and Aruin, 2015) and in a reaching task while standing (Saito et al., 2014). Improvements were invariably associated with an earlier recruitment of the APAs in lower limb muscles.

#### APA can be specifically affected in neural diseases: hints to clinical diagnostics

Postural and focal movements can be de-coupled in case of nervous diseases. There seems to be no single “APA organ” within the central nervous system: rather, many CNS structures are involved. These include hemispheric cortex, cerebellum, basal ganglia, brain stem, and spinal cord (for a quick overview, see Discussion in Ioffe et al., 2007).

**Figure 13:**
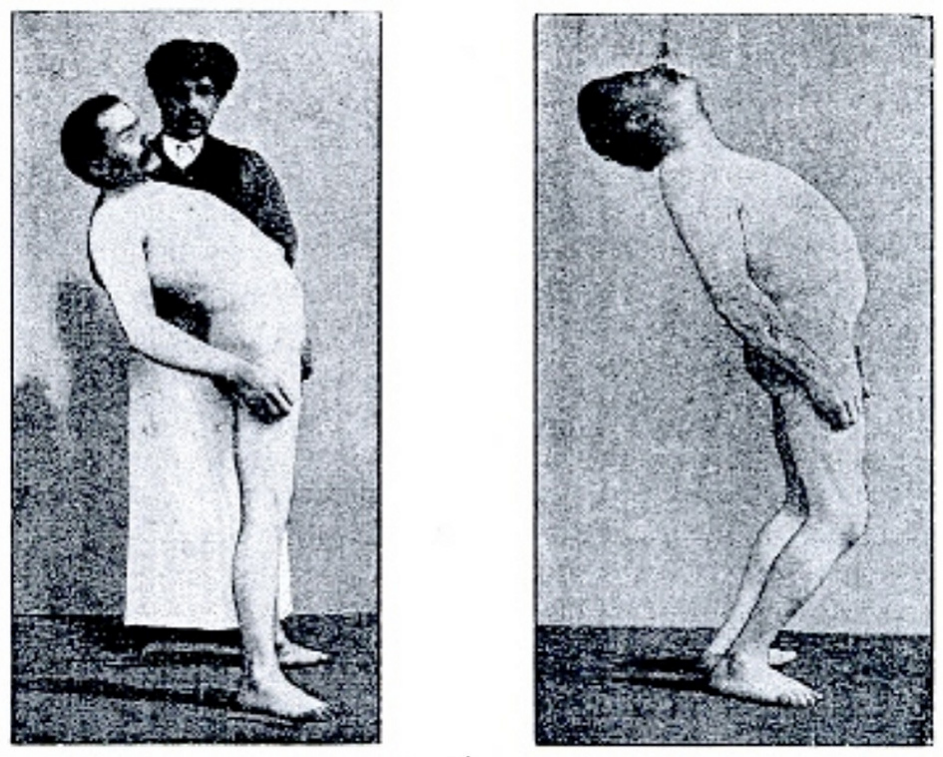
**A cerebellar patient (left panel) is requested to lean backward (focal movement) while standing**. If not supported by the assistant, he will fall. The right panel shows the correct movement, in which knee flexion (APA) precedes the trunk extension, so that the body center of mass will be projected within the base of support (“cerebellar asynergy,” after Babinski, 1899). This established clinical finding was confirmed by later, refined neurophysiologic research. Yet, after nearly 120 years this still remains one of the brightest demonstrations that the postural component of a voluntary moment can be impaired independently from its focal component.

The cerebellum is certainly a key node of the network of postural control (Ioffe et al., 2007). The fact that cerebellar damage entails “postural” consequences was already acknowledged, as shown in Figure 13 by a famous Babinski's example of a “postural” alteration after cerebellar lesion leaving the “focal” movement unaffected (Babinski, 1899). Cerebellar lesions may selectively impair also the APAs subtending within-limb movements. For instance, in patients with cerebellar ataxia, simple brisk movements of the finger are associated with alterations of the APAs engaging proximal arm muscles (Bruttini et al., 2015; Cavallari et al., 2016).

Recent research also showed that the lesion of many other cerebral structures (e.g., of the supplementary motor area (Viallet et al., 1992) or of the basal ganglia (Viallet et al., 1987) can lead to selective alterations of APAs, thus affecting within-limb and/or body-ground chaining. With respect to the “easy” and “difficult” hand-foot coupling described above, it has been shown that after hemispheric stroke, the “difficult” coupling is selectively impaired (or even impossible) on the unaffected side, which retains an otherwise skilled motricity (Baldissera et al., 1994). The frame of APAs physiology offers an explanation.

As illustrated in Section Role of APAs in Differentiating ISO vs. ANTI Coupling Modes in Other Types of Limb Movements, APAs may well-spread contralaterally to a focal movement; further, the successful completion of the APA “arborization” is a pre-requisite for the subsequent contraction of focal muscles (Cordo and Nashner, 1982; Brown and Frank, 1987). Hence, any impairment of APA commands on the paretic side may impair or even prevent a focal movement on the unaffected side. Not surprisingly, in hemiparetic patients, strength and coordination are affected on the “healthy” side, too (Santos et al., 2016). While overt postural disturbances may not appear, yet the hand-foot coupling test may reveal that they stand subliminal in the background. Therefore, asking for anti-phase coupling of the unimpaired hand and foot can be proposed as a simple, yet highly sensitive bedside test of hemispheric damage. The test is not specific to hemispheric lesions, however. All of the above considerations stimulate clinical reasoning. The studies on APAs in cerebellar patients suggest that ataxia can be seen as a typical consequence of APAs disorders, not less than of the programming of focal movements. Limb ataxia (commonly evidenced by dismetry in the classic index-to-nose or heel-to-knee tests), the incapacity to maintain a stable level of isometric force (“dys-stenia,” Tesio, 2010), as well as trunk ataxia with balance deficits can all be interpreted as primarily caused by APAs disturbances. The subject knows what the focal movement should be but is unable to avoid position and/or force errors due to altered APAs.

The capacity of disease to affect selectively focal rather than postural movements is demonstrated by the case of survival of APAs after loss of its related focal movement. This observation is common in clinical practice but only exceptionally reported in the literature. For instance, ischemic or anesthetic block of the hand does not abolish APAs in proximal muscles (Bruttini et al., 2014). Brachial plexus lesions may entail the complete loss of shoulder abduction, yet the “luxury” of a fixation chain crossing the midline during a voluntary effort is preserved. Thus, the very existence of APA warrants the presence of genuine “will,” thus providing a useful clinical sign. Fixation chains can only be rarely lost: they may disappear in case of cognitive deficits (e.g., hemineglect or apraxia), chronic “learned non-use” in deep-seated palsies (Taub et al., 2014) or conditions such as simulation and conversion disorders (Tesio and Colombo, 1992).

#### APA can be selectively trained in sports and rehabilitation medicine

Motor training science (Magill and Anderson, 2014) has long acknowledged that “coupling” some movements is much more difficult to be trained and learned, compared to other. For instance, teaching how to de-couple the upper limbs in bimanual activities, usually a difficult challenge, is made more effective by dedicated training techniques (Walter and Swinnen, 1994).

Although training a “voluntary” movement is usually explicitly addressed to its focal component, yet it entails an unconscious and indirect training of its postural component (Mouchnino et al., 1992; Ioffe et al., 2007). As a rule, training can be addressed to the desired focal movement (explicit training) or another actual or imagined movement to which the desired one is unconsciously associated (implicit training). This holds also for skilled exercises improperly claimed to directly train the APAs (Aruin et al., 2015; Kanekar and Aruin, 2015). Actually, neither approach targets explicitly the APAs. Specific APA training paradigms are still missing. First, the APA chains subtending the normal voluntary movements must be predicted: although they mostly spread along the plane of the focal movement (Gabbett and Masters, 2011; Bruttini et al., 2014) a detailed “atlas” of expected APAs in various motor tasks is far from being developed. Second, APAs disorders usually consist in incomplete chaining and/or delayed timing/phasing between focal and various postural muscles. The between-muscle delays are in the order of tenths of milliseconds, depending on the muscles involved. In a clinical environment, a very skilled palpation of tendon tension can often succeed in capturing such between-muscle delays. A much easier and sharper temporal discrimination, in the order of few milliseconds, can be reached, both by patient and therapist, by binaural listening of sharp tones generated by transduction of the surface EMG from a focal and a postural muscle, and separately sent to the right and left ears (Tesio et al., 1996). This form of augmented feedback can be said to explicitly target the APAs and to provide a “knowledge of result” fostering the learning of the correct APA (Schmidt and Lee, 2005).

Despite the examples given above, the diagnosis and the treatment of APAs' deficits, both in sports and rehabilitation sciences, is still at the frontiers of physiology and represent a promising field of research.

## Author contributions

All authors contributed in collecting the literature, critically analyzing it, and writing the manuscript. All authors approved the final version and agree to be accountable for all aspects of this work.

### Conflict of interest statement

The authors declare that the research was conducted in the absence of any commercial or financial relationships that could be construed as a potential conflict of interest.

## Footnotes

^1^Throughout this review the movement direction will always be referred to external coordinates.

## Abbreviations

Muscles: ECR: Extensor Carpi Radialis
FCR: Flexor Carpi Radialis
rPM: right Pectoralis Major
lPM: left Pectoralis Major
lFCR: right Infraspinatus
lIS: left Infraspinatus
rAD: right Anterior Deltoid
lAD: left Anterior Deltoid
rPD: right Posterior Deltoid
rLD: right Latissimus Dorsi
lPD: left Latissimus Dorsi
rES: right Erector Spinae
lES: left Erector Spinae
rIC: right Ischiocruralis
lIC: left Ischiocruralis
SOL: Soleus
TA, Tibialis Anterior. Neurophysiologic and mechanical variables: CMAPs: Compound Muscle Action Potentials
TMS: Transcranial Magnetic Stimulation
CoP: Center of Pressure
Δ$\dot{\text{V}}$O_2_: oxygen uptake
ΔΦ: interlimb relative phase
SDΔΦ: Standard Deviation of the ΔΦ variability.
