# Supplementary material for: APAs Constraints to Voluntary Movements: The Case for Limb Movements Coupling
Source: Front Hum Neurosci. 2017 Mar 31;11:152. doi: 10.3389/fnhum.2017.00152 (PMC5374888; doi:10.3389/fnhum.2017.00152)
Supplement: Supplementary file 1 [file Presentation1.PDF]

Presentation 1 A model for the phase transition from the non-preferred to the preferred coupling revisited

No direct experimental evidence is available on the intimate neuronal mechanism responsible for the sudden phase transition from the non-preferred to the preferred coupling. So far, this feature has been interpreted in the reference frame of the Haken-Kelso-Bunz model (Haken et al. 1985; see also Swinnen 2002), in which the interlimb relative phase ($\Delta\Phi$) is determined by the relative power of two immaterial attractors, located at $\Delta\Phi = 0°$ (mirror symmetrical coupling) and $\Delta\Phi = 180°$ (isodirectional coupling), respectively (in this model, as well as in most of the literature on upper limb coupling, the phase of cyclic movements is referred to the body plane of symmetry, not to absolute coordinates). In this model the phase behaves like of a ball rolling in a potential energy profile with two adjacent basins of attraction, representing the metastable state ($\Delta\Phi = 180°$, local minimum potential) and the stable state ($\Delta\Phi = 0°$, absolute minimum potential), respectively. A potential barrier separates the two basins, allowing $\Delta\Phi$ to remain anchored to one or the other attractor. In this frame, the intrinsic variability of the potential is reflected into the variability of the relative phase, SD$\Delta\Phi$, as it may be visualised by the ball oscillations.

The potential profile is described by the function $V(\Delta\Phi) = -a\, cos(\Delta\Phi) - b\, cos(2\Delta\Phi)$, which described the tendency of $\Delta\Phi$ to change as a function of its own value. The ratio *b/a* defines the height of the potential barrier between the basins. The potential barrier is assumed to decrease when the cycling frequency increases. This, in turn, would increase the amplitude of the ball oscillations (the $\Delta\Phi$ variability) until it becomes sufficient to push the ball falling from the highest ($\Delta\Phi = 180°$) to the lowest ($\Delta\Phi= 0°$) basin of attraction and $\Delta\Phi$ to reverse.

The linkage disclosed between APAs and coupling stability now allows to associate this purely descriptive model with some definite physiological mechanisms and parameters. For instance, applying the model to coupling of horizontal arm movements and expressing $\Delta\Phi$ in absolute coordinates, APAs might be imagined as an attractor at $\Delta\Phi = 180°$ (mirror symmetrical, ANTI coupling) thus being described by the term $a\, cos(\Delta\Phi)$. In turn, the voluntary commands, which can attract the phase either to 180° (ANTI coupling) or to 0° (ISO coupling) and are therefore either favoured or contrasted by the APAs, might be described by the second term, $-b\, cos(2\Delta\Phi)$.

In ANTI coupling, in which APAs and voluntary commands have the same sign (both are excitatory or inhibitory) the two attraction poles coincide and the relative phase $\Delta\Phi$ is stable at 180°. In ISO, instead, the voluntary commands to one arm and the APAs linked the voluntary movements of the other arm have opposite sign, so that $\Delta\Phi$ can be maintained at 0° (ISO) only if the potential barrier *b/a* is high enough to avoid the ball dropping in 180° basin. APAs suppression (gating) may thus be the factor that in ISO coupling maintains *a* to a minimum and the ratio *b/a* high enough to keep $\Delta\Phi = 0°$. In this view, the dynamics of APAs gating would represent the link between the movement frequency and the size of the potential barrier; and gating default would be the factor causing the ball to fall in the deepest potential basin and $\Delta\Phi$ to reverse.

The same model can apply to arm parasagittal movements, as well as to hand-foot coupling, after phase-reversing the APAs attractor pole.

Haken, H., Kelso, J.A., Bunz, H. (1985) A theoretical model of phase transitions in human hand movements. *Biol. Cybern*. 51, 347-356.

Swinnen, S.P. (2002) Intermanual coordination: from behavioural principles to neural-network interactions. *Nat. Rev. Neurosci.* 3, 348-359.
