# Supplementary material for: APAs Constraints to Voluntary Movements: The Case for Limb Movements Coupling
Source: Front Hum Neurosci. 2017 Mar 31;11:152. doi: 10.3389/fnhum.2017.00152 (PMC5374888; doi:10.3389/fnhum.2017.00152)
Supplement: Supplementary file 2 [file Presentation2.PDF]

**Presentation 2 Fitting the hand and foot oscillations by the pendulum equation**

The input-output transfer function of an oscillating pendulum driven by a sinusoidal force describes the changes occurring in the *gain* [the ratio between the output (movement) and the input (force)] and *phase* [between the force and movement sine-waves] when the oscillation frequency is increased. The shape of the transfer function is determined by the values of the system's mass, stiffness and viscosity. In the case of limbs oscillations, the undetermined correspondence between the surface EMG signals and the muscular force makes the estimate of the gain unreliable. Instead, the force-to-movement phase-relations of a pendulum of a given mass is independent from the peak force injected and only depends on the system stiffness and viscosity. The relation is described by the equation $\phi = arctan\frac{-\Omega \cdot V}{K - I \cdot \Omega^2}$, where $I$ is the inertial momentum, K the torque stiffness, V the torque viscosity and Ω the pulsation, 2π*F*. In this model, when the oscillation frequency increases the output phase-delay grows sigmoidally from 0° to 180°, crossing 90° at the resonance frequency, $rF = \sqrt{K/I}$, at which the inertial and elastic resistance are equal and phase opposite, so as to cancel each other. An increase of the inertial momentum mainly shifts the curve (and *rF*) to the left, with small effects on the curve slope. The opposite is obtained when increasing the stiffness parameter (*K*). Finally, changing the system's viscosity has no effect upon *rF* but modifies the slope of the curve, which becomes shallower when V gets larger.

In each subject, fitting of the experimental values of the phase-relation of the hand oscillation with the pendulum model was performed after substituting the parameter *I* in the model equation with the inertial momentum of his/her hand, added with the momentum of the supporting platform. Values of the lumped stiffness and viscosity (*K* and *V*, respectively) which fitted the experimental points best, as well as the resonance frequency (*rF*) of each curve, were computed and utilised for statistical comparisons.

***Intrinsic frequency-response of the hand***

In resting conditions the equilibrium position of the hand prone is located at about -50° flexion from the horizontal forearm, around 10°-15° from the end point of the wrist flexion. The hand oscillations could thus be maintained all above the equilibrium position (*high range,* **Figure 1A**) or could be centered around the equilibrium position (*central range,* **B**). Most subjects choose the high range spontaneously and the central range only if specifically requested. In the *high range,* the hand oscillations are sustained by the sole extensor muscles, which first lift the hand and then brake the ensuing flexion driven by gravity and elastic recoil. In this case the force-movement phase-difference is measured between the onset of the EMG burst in ECR and the onset of the hand extension (EMG → ext ΔΦ). In the *central range* both flexor and extensor muscles are recruited, and switching between antagonists occurs at the equilibrium crossing. The force (EMG) onset is then correlated to the hand equilibrium position (EMG → eq ΔΦ).

The hand phase-responses in the *central* and in the *high* ranges are well fitted (**C**), over a substantial frequency range, by the pendulum equation and do not substantially differ from each other (Esposti *et al.,* 2005)

Inertial loading of the hand induces a faster decay of the hand phase response (Baldissera *et al.,* 2000). Conversely, hand elastic loading provokes a shift of the phase-curve to the right with respect to the unloaded condition. In both cases, after adding to the model equation the *I* or the *K* values of the applied loads, the changes conform to the model predictions. When hand loading increased the system inertia, fitting the model equation required a higher value for the model stiffness, suggesting that a reactive increment of the total stiffness of the limb had occurred, justified by the compensatory increase in the muscle "active state" (Hunter and Kearney 1982).

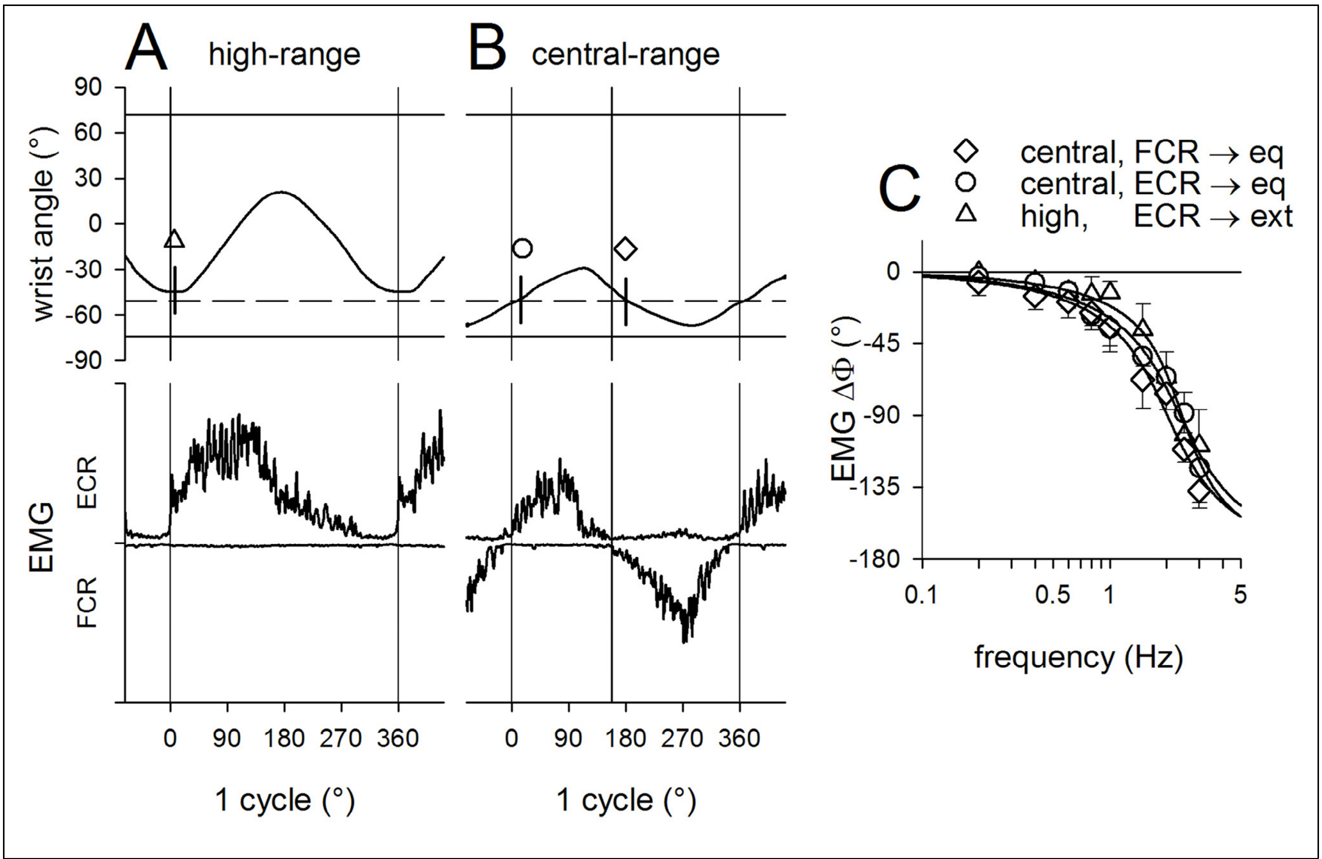


**Figure 1**. Activity in antagonist forearm muscles during cyclic flexion-extensions of the prone hand, performed at 0.4 Hz over two different ranges of the joint excursion. **A** and **B**: wrist angle (upper traces) and rectified integrated EMG from *ECR* and *FCR* (lower traces). FCR EMG inverted. Horizontal solid lines, anatomical limits of wrist excursion; dashed lines, joint passive equilibrium position. Vertical lines, onset of EMG recruitment. Vertical segments: joint position at the EMG onset of the extension phase (A) or at the crossing of the equilibrium position (B). **C**: Hand frequency-response measured in the high range (ECR →ext) and in the central-range (ECR→eq and FCR→eq curve). Solid lines; best-fit curves of the pendulum equation. *Reproduced from Baldissera et al. (2005) © Springer-Verlag Berlin Heidelberg 2005, with permission of Springer.*

### *Intrinsic frequency response of the foot*

Foot oscillations can also be performed across the equilibrium position (*central-range*) as well as all above (*high-range*) or all below (*low-range*) the equilibrium. As for the hand, the pendulum equation fits the foot phase-response if the correct homology between the onset of the prime movers EMG and the oscillation (either the onset or the equilibrium crossing) is respected (Baldissera *et al.,* 2004). Phase responses are similar in all three oscillations ranges and when the frequency increases they show a slower decay and a higher value of the ratio $\sqrt{K/I}$ (the resonant frequency) than the corresponding responses of the hand, indicating that the larger mass of the foot is naturally associated with an even large increase of the active stiffness.

## References

Baldissera, F., Borroni. P., Cavallari, P. (2000) Neural compensation for mechanical differences between hand and foot during coupled oscillations of the two segments. *Exp. Brain. Res.* 133, 165–177.

Baldissera, F., Cavallari, P., Esposti, R. (2004) Foot equilibrium position controls partition of voluntary command to antagonists during foot oscillations. *Exp. Brain. Res.* 155, 274–282.

Esposti, R., Cavallari, P., Baldissera, F. (2005) Partition of voluntary command to antagonist muscles during cyclic flexion–extension of the hand. *Exp. Brain. Res.* 162, 436–448.

Hunter, I.W., Kearney, R.E. (1982) Dynamics of human ankle stiffness: variation with men ankle torque. *J. Biomech.* 15, 747-752.
