# Supplementary material for: APAs Constraints to Voluntary Movements: The Case for Limb Movements Coupling
Source: Front Hum Neurosci. 2017 Mar 31;11:152. doi: 10.3389/fnhum.2017.00152 (PMC5374888; doi:10.3389/fnhum.2017.00152)
Supplement: Supplementary file 3 [file Presentation3.PDF]

Presentation 3 The neural and mechanical delays of the hand and foot oscillations from the clock signal are invariant when the limbs are oscillated in isolation or when they are coupled.

Analysis of the *clock-mov* delay also included the measurement of its two components, the *neur* delay and the *clock* delay. Three interconnected signals were considered (**Figure 1**): 1) the sinusoidal course of the synaptic input to flexor and extensor motoneurones *(MnIn)*, estimated as that of the sine-wave interpolating the onset of the successive EMG bursts in the antagonists (see Baldissera et al 2006); 2) the ensuing EMG activity in the related muscles and, 3) the resulting movement (*mov*).

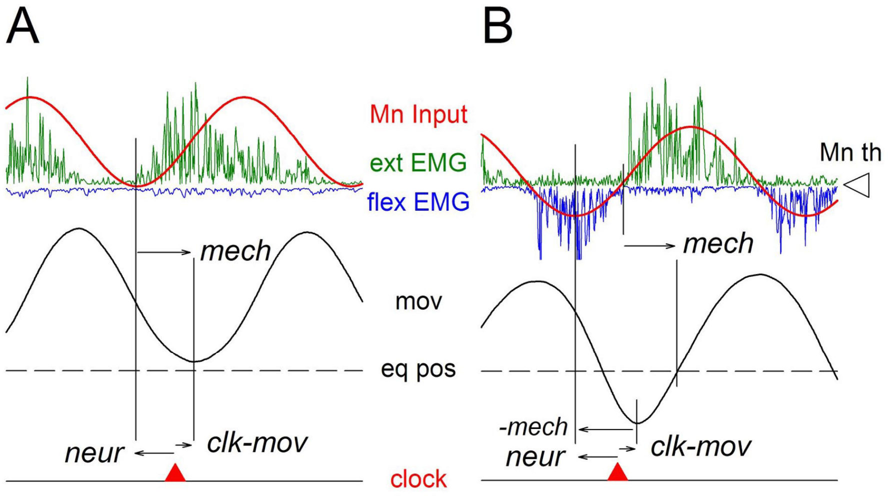

Figure 1. Relevant phase measurements used for analysing the synchronism of the limb oscillations with a clock signal. Limb oscillations all above (**A**) and across (**B**) the equilibrium position. Uppermost traces: rectified EMG from extensors (green trace) and flexors (blue trace, reversed) muscles; sine-wave synaptic input to motoneurones (*MnIn*, red solid line) supposed to drive the limb oscillation. *MnIn* has the same course, but is sign-reversed, in flexors and extensors. *Mn th* (open arrowhead): motoneurones threshold level. Lower traces: ensuing movement (*mov*), limb passive equilibrium position (*eq pos*, dashed line) and clock signal (upward red triangle). Horizontal arrows mark the *clk-mov*, the *neur* and the *mech* delays. *From Baldissera et al.2006, BMC Nurosci. 7,70.*

Three phase delays were then measured, i.e., i) the *clk-mov* delay, between the rhythmic clock beat and one peak of the limb oscillation; and its two components: ii) the *neur* delay (actually an anticipation) between the clock signal and the peak of the *MnIn* sine-wave (which also includes the negligible conduction time in peripheral motor axons); and iii) the *mech* delay (caused by the mechanical impedance of the limb) between the onset of the motoneuronal disharge (EMG burst) and the corresponding point of the movement. The three delays are illustrated for the two situations in which the limb movements are all above (A) or across (B) the joint equilibrium position. The *clk-mov* curves in **Figure 2A** (both limbs unloaded) show that the movement peak maintains an almost constant phase relation with the metronome beat (negative values: movement delay) both when the limbs are moved in isolation (green solid and dashed lines) or when they are isodirectionally coupled (hand: blue circles, foot: red triangles).

Figure 2B illustrates how the movements phase lock is achieved. The two *mech* curves (filled circles) describe the mechanical phase-response of each oscillating segment and are well fitted by the pendulum equation, the resulting resonance frequency (*rF*) being significantly lower for the hand than for the foot.

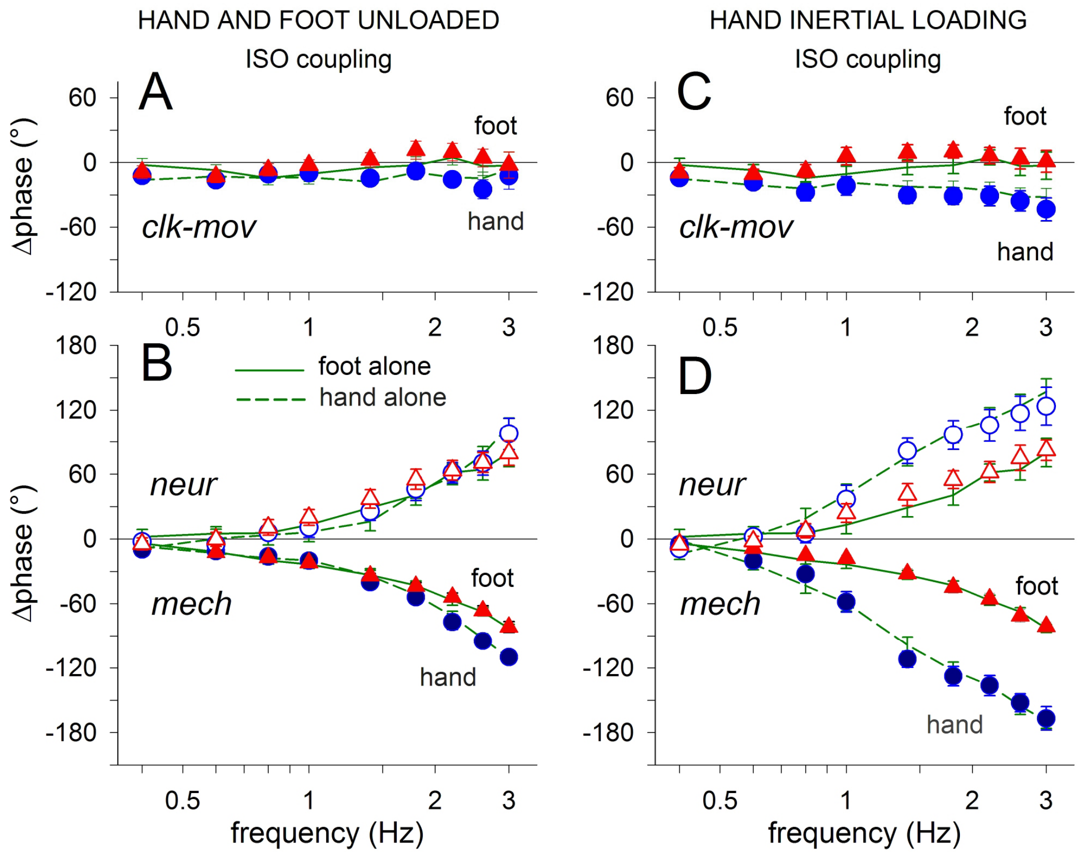

Figure 2. **A**. When both limbs are unloaded the *clk-mov* delay of each limb, no matter whether the limb is isolated or coupled, remains nearly constant over the whole frequency range. **B**: *mech* curves (filled symbols, same as in A) trace the corresponding increase of the EMG-movement delay, while *neur* curves (open symbols) show the simultaneous phase-advance of the EMG activation with respect to the clock signal, which maintains the *clk-mov* delay constant (panel A). **C**, **D:** same relations as in A and B but obtained after applying an inertial load to the hand. Loading induced the expected increase of both the *mech* delay and the *neur* advance of the hand (D). The latter is however insufficient to completely compensate for the *mech* lag, so that the *clock-mov* delay of the hand increases as the frequency is raised (C). In each limb all the frequency-relations measured during separate and coupled movements strictly superimpose.

In either extremity the course of the *neur* curves (open symbols) virtually mirrors the corresponding *mech* curves (filled symbols), showing that the progressive advance of the EMG onset with respect to the metronome beat is calibrated to just quantitatively compensate for the increasing lag of the *mech* curve, thus ensuring in each segment the maintenance of a steady *clk-mov* delay over the entire frequency range.

It is also well evident that the *mech* and *neur* curves referring to the coupled movements (symbols) are practically superimposed to those referring to movements of the isolated limbs (green lines), with no significant difference between their best-fit functions, even after enhancing the mechanical difference between the limbs, by separately connecting the hand **(**Fig. 2 C and D**)** or the foot (not illustrated) to an inertial load. Loading of one limb is effective in dividing the *clk-mov* curves of the two segments from each other, but it is ineffective in separating the phase curves of the limbs when moved alone or coupled together.

References

Baldissera, F.G., Cavallari P., Esposti. R. (2006) Synchrony of hand–foot coupled movements: is it attained by mutual feedback entrainment or by independent linkage of each limb to a common rhythm generator? *BMC Neurosci.* 7, 70.
