# Supplementary material for: APAs Constraints to Voluntary Movements: The Case for Limb Movements Coupling
Source: Front Hum Neurosci. 2017 Mar 31;11:152. doi: 10.3389/fnhum.2017.00152 (PMC5374888; doi:10.3389/fnhum.2017.00152)
Supplement: Supplementary file 4 [file Presentation4.PDF]

Presentation 4.0 Control of joint position by a kinaesthetic feed-back

Results presented in the review Section 2 suggest that during coupled movements each limb controls its own position with respect to the common rhythm generator through a “private” kinaesthetic feedback. In this regard, analysis of the EMG-movement phase relations in the hand and foot gave some useful clues to the operation properties of such a mechanism.

During rhythmic oscillations of the hand or foot, switching from one mover to its antagonist always occurs when the moving limb crosses its passive equilibrium position (Baldissera *et al.* 2005). This would imply that every time the equilibrium position is modified - e.g., by loading the limb or by changing its position with respect to gravity - the nervous system must reset this information to properly rearrange the antagonists alternation.

Experimental manipulation of the hand passive equilibrium validates this assumption. When the hand, supported by a rotating frame, is semi-prone with the palm facing the body midline, wrist flexion-extension is performed in the horizontal plane. In this way the effects of gravity on movements are removed and the elastic recoil from either extreme of the joint excursion vanishes over a central range, so that the hand is in equilibrium not in a single point but over an entire sector (grey band in **Figure 1A**)

If the hand oscillates around the *equilibrium sector* each antagonist muscle is recruited when the hand enters that range and meets a dissipative resistance to be overcome: therefore in a different hand position for the two muscles and movement directions.

Despite the non-linearity introduced by the equilibrium range, the pendulum equation well fits the ECR→eq and FCR→eq phase curves (**Figure 1D**) as in the case of the hand prone. Applying an adequate and constant frictional resistance to the wrist rotation creates a broad equilibrium range (grey band in **Figure 1B**), within which the entire hand oscillation can be confined. In this situation concentric muscle contraction is needed throughout the entire movement and switching between antagonists occurs at the movement reversal points, in coincidence with the inversion of the frictional resistance.

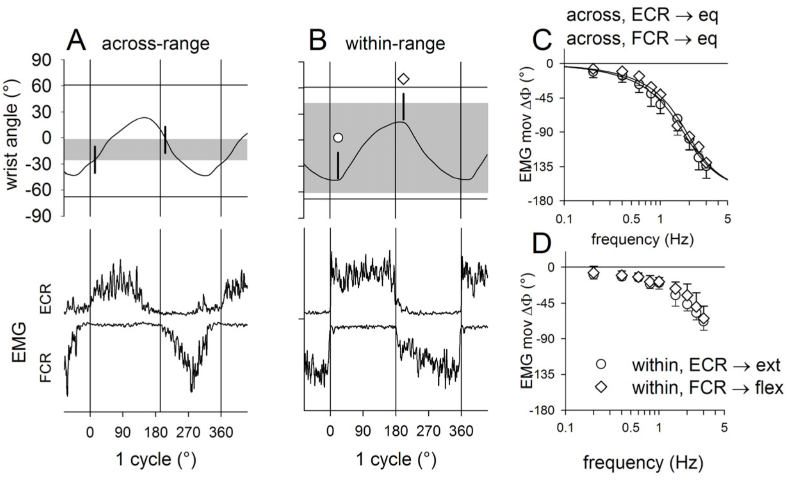

Figure 1. Different patterns of muscle activity in cyclic (0.4 Hz) flexion-extension of the hand in the horizontal plane (hand semi-prone), performed across a narrow (**A**) and within a large (**B**) equilibrium range. Uppermost plots: wrist angular excursion. Horizontal continuous lines: limits of the wrist excursion. Grey band: wrist equili-brium range. Small vertical bars: joint position homologous to the EMG onsets Lowermost plots: rectified and integrated EMG from ECR and FCR (inverted traces). Vertical lines, onset of the EMG activity in the two antagonists;. **C**: hand frequency response measured in the across-range (ECR → eq and FCR → eq curves, circles and diamonds, respectively). Solid lines: curves of a pendulum model that fit each data set best. **D:** frequency responses measured in the within-range (ECR → ext and FCR → flex curves, circles and diamonds, respectively)

In summary, despite the differences in the equilibrium position and in the type of mechanical resistance, in any of the examined situations each forearm muscle is recruited when a resistance increase is met and its force is needed to continue the programmed movement. It may be then

argued that encountering a new resistance, of whatever nature and at any time, will slow the ongoing movement and create a mismatch between the actual hand position, encoded by the kinaesthetic afferents, and the intended position, hypothetically encoded by the voluntary central command. By monitoring this error, the CNS may then build up the convenient motor correction.

Knowledge of the phase-response of the hand and foot oscillations allowed to formalize the above concepts in a closed-loop control system (Esposti et al. 2005, 2007), able to provide coincidence of the actual with the intended position of the limb.

A model for feedback control of joint position

The model is composed by: 1) a mechanical block (pendulum model) reproducing the simplified musculoskeletal properties of a monoarticular limb segment, capable of flexion–extension movements; 2) a simple neural network, (Baldissera et al. 2004) which transmits a central motor command to motoneurones and splits the activation between antagonist muscles; 3) a feedback line that conveys kinaesthetic information on the limb position to be compared to the central command.

Functioning of this control system rests on the assumption that during voluntary movement the control variable (the central command) encodes the time profile of the intended joint angular

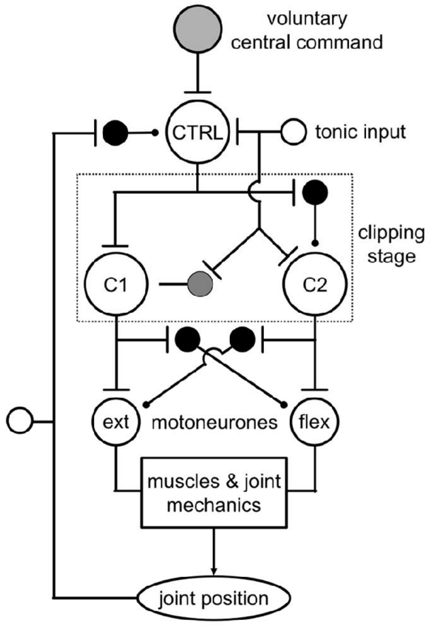

Figure 2. Model of a feedback controller of the joint position. The neural network includes only those elements which are indispensable for obtaining the desired performance. Values for the model mechanical parameters *K* and *V* for each limb and load combination were derived from fitting the experimental *mech* curves with the pendulum equation. The moment of inertia, *I*, was the sum of moments of the limb, the rotating platform and, when applied, the inertial load.

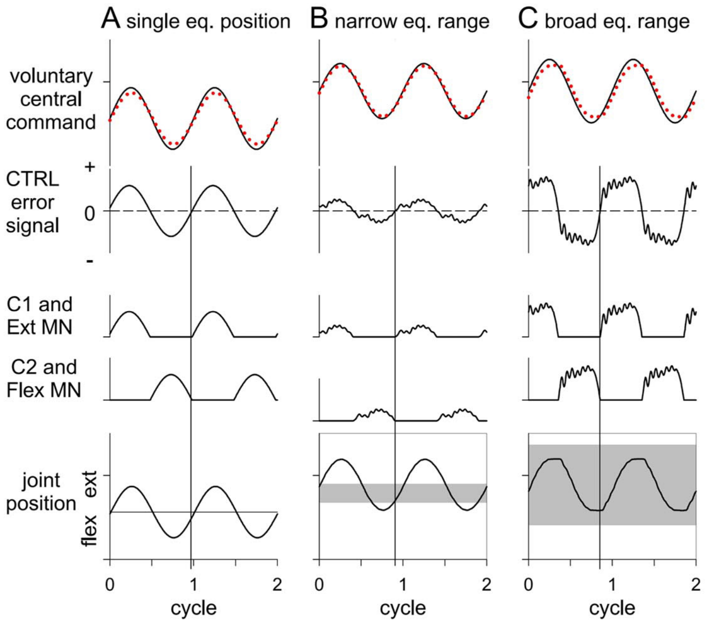

Figure 3. Hand oscillations (0.4 Hz) generated by the model in Figure. 2. Three different mechanical conditions: **A**, hand prone oscillating in the vertical plane, single equilibrium position; **B**, hand semi-prone oscillating in the horizontal plane, narrow equilibrium range; **C**, same as in B, oscillations confined within an enlarged equilibrium range. Equilibrium position and ranges marked by a line (A) and grey bands (B and C) in the joint position traces. The latter are also superimposed by red dots on the trace of voluntary central command for comparison. Sub-threshold modulation in clipping neurones and motoneurones is not illustrated. Because of the long oscillation period, effects of conduction delays between the different elements of the network are not appreciable. When the CTRL error signal rises above zero (vertical lines) C1 starts and C2 stops firing. Depending on the mechanical context, C1 activation is temporally aligned with different points of the limb movement, just as it occurs for EMG onset in real experiments (Fig. 1).

position, without specifying muscle activation, which is dimensioned by the controller neurones (see Todorov, 2000). Further assumption are that all neurones are kept at threshold by some not

defined mechanism; that signal transmission is linear; and that excitatory and inhibitory synapses are equipotent.

Briefly, the model control element (CTR in **Figure 2**) receives two independent synaptic inputs: 1) an excitatory voluntary central command (encoding the time sequence of the intended joint angular positions) and 2) a feedback kinaesthetic signal (encoding the actual joint position), sign-reversed by an inhibitory interneurone. When the two signals are equal, the CTRL output (i.e., the error signal) is = 0. When their sum is ≠ 0, an error signal is generated, linearly amplified, and dispatched to two clipping neurones with excitation (C1) or inhibition via an interposed interneurone (C2). If the error signal is positive (feedback signal lower than control signal), C1 fires and activates one motoneurone pool (e.g. extensor). Reciprocally, when the error signal is negative, C2 activates the antagonist pool (e.g. flexor). The sign and size of the error signal will thus determine the direction (agonist vs antagonist) and the amount of motor activation. In this way, the amplitude and phase of the motoneurone input depend, through a proportional gain (μ), on the difference between the sinusoidal central drive (intended movement) and the sinusoidal afferent signal (actual movement), thus continuously correcting the position discrepancy. After implementing the model mechanical block with parametral values pertaining to the hand or foot, introducing realistic values for the conduction delays and assuming a linear summation of the neural excitation and inhibition - the closed-loop control system adequately simulates the different activity distribution between antagonists observed both in static limb postures and during low-frequency limb oscillations in the presence of both a single equilibrium position and a narrow or large equilibrium range (**Figure 3**)

The possible correspondence of elements of the control circuit with some known CNS structures are discussed in Esposti et al. (2005 and 2007). In particular, firing of all corticospinal neurones (Cheney and Fetz 1980, Cheney et al 1982) correlates with the joint torque in one specific direction, indicating that the proposed integration of the feedback position signal with the motor command should occur upstream to that site.

After modifying the gain of the CTRL output, this same model can also maintain the synchronism between a rhythm generator and the oscillation of a limb during sinusoidal activation, as requested by the "private" kinaesthetic feedback hypothesis. A proportional gain (μ) was indeed unsuited to satisfactorily approach the limbs behaviour, especially in the loaded condition (see **Figure 4**, green dashed lines). Instead, adding to the proportional gain (μ) also a derivative (τd) and an integrative (τi) gain components (proportional, integrative and derivative, PID, controller) the model can accurately fit the experimental *clk-mov* and *neur* data (**Figure 4** red continuos lines) over the tested frequency range (0.4–3.0Hz), even after extra loading (Esposti etal. 2007).

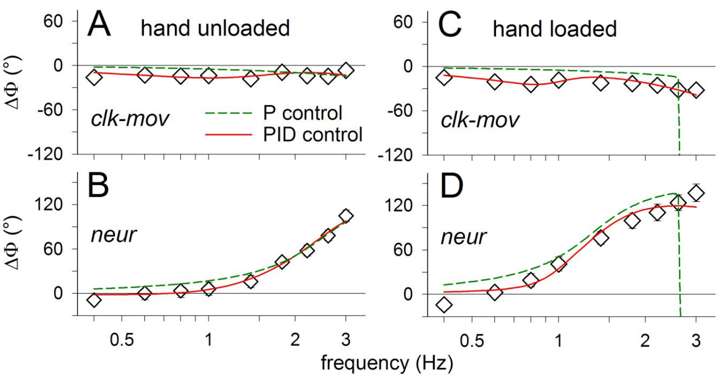

Figure 4 *Clk-mov* and *neur* curves of the hand as simulated by a feedback position controller. The dashed green lines describe the response of the Proportional (P) model version, the continuous red lines the response of the Proportional, Integrative and Derivative (PID) version. Symbols reproduce the experimental data from which the model parameters were taken. Note the much higher accuracy of the PID model in describing the hand behaviour, even. after loading. A similar accuracy was obtained for the foot.

Altogether, the above simulations seem to confirm that the experimental data and the functional mechanisms conceived are congruent with each other in suggesting that each limb is equipped with a closed-loop, PID position controller, which provides matching of the actual limb position with the

position intended by the central command entrained by the clock beat. If so, when movements of the hand and foot are coupled, the position controller of each limb is *per se* sufficient to keep the limbs synchronised with each other without need for any interlimb information exchange, both in iso- and antidirectional coupling and even when the mechanical disparity between the moving segments is heavily enhanced.

Interestingly, by dissecting the variance of movement cycle duration into a *clock* and a *motor* components, Wing and Kristofferson (1973) distinguished two corresponding (*clock* and *motor)* levels in the control of rhythmic limb oscillations. On the same line, Turvey et al.(1989) later showed that during bilateral wrist pendulum oscillations, asymmetries of the limbs loading only affect the correlation between the right and left *motor* variances, while the two *clock* variances were still correlated. Conversely, the contrast of phase symmetry (iso- vs antidirectional oscillations) affected the correlation between the *clock* variances but not the right and left motor variances (Turvey et al 1986). In summary, during coupled oscillations the operations at the motor level were found to be sensitive to limb loading but indifferent to the coupling modality (iso- or antidirectional), i.e., to share the same properties as the position control modelled here.

References

Baldissera, F., Cavallari, P., Esposti, R. (2004) Foot equilibrium position controls partition of voluntary command to antagonists during foot oscillations. *Exp. Brain. Res.* 155, 274–282.

Cheney, P.D., Fetz. E.E. (1980) Functional classes of primate corticomotoneuronal cells and their relation to active force. *J. Neurophysiol*. 44, 773–791

Cheney, P.D., Kasser, R., Holsapple, J. (1982) Reciprocal effect of single corticomotoneuronal cells on wrist extensor and flexor muscle activity in the primate. *Brain Res* 247, 164–168

Esposti, R., Cavallari, P., Baldissera, F. (2005) Partition of voluntary command to antagonist muscles during cyclic flexion–extension of the hand. *Exp. Brain. Res.* 162, 436–448.

Esposti, R., Cavallari, P., Baldissera, F. (2007) Feedback control of the limbs position during voluntary rhythmic oscillation *Biol. Cybern.* 97, 123–136

Todorov, E. (2000) Direct cortical control of muscle activation in voluntary arm movements: a model. *Nat. Neurosci,* 3, 391–398

Turvey, M.T., Rosenblum, L.D., Schmidt, R.C., Kugler, P.N. (1986) Fluctuations and phase symmetry in coordinated rhythmic movements. *J. Exp. Psychol. Hum. Percept. Perform.* 12,564-583.

Turvey, M.T., Schmidt, R.C., Rosenblum, L.D. (1989) 'Clock' and 'motor'components in absolute coordination of rhythmic movements. *Neuroscience* 33,1–10

Wing, A.M., Kristofferson, A.B. (1973) Response delays and the timing of discrete motor responses. *Percept Psychophys*. 14, 5–12
